# Glial cytokine modulation improves sleep and circadian disruption in female SAA knock‐in mice of Alzheimer's‐related pathology

**DOI:** 10.1002/alz.71314

**Published:** 2026-03-30

**Authors:** Teresa Macheda, Margaret R. Hawkins, Carrie E. Johnson, Madison G. Lapid, Haleigh R. Whitlock, Savannah M. Shepard, MaKayla F. Cox, Kelly N. Roberts, Leke Bytyqi, Heather M. Hash, Omar A Abou El‐Ezz, Mohammed Abou El‐Ezz, Katherina Kohler, Sridhar Sunderam, Bruce F. O'Hara, Linda J. Van Eldik, Michael P. Murphy, Marilyn J. Duncan, Adam D. Bachstetter

**Affiliations:** ^1^ Department of Neuroscience University of Kentucky Lexington Kentucky USA; ^2^ Spinal Cord and Brain Injury Research Center University of Kentucky Lexington Kentucky USA; ^3^ Sanders‐Brown Center on Aging University of Kentucky Lexington Kentucky USA; ^4^ Department of Molecular & Cellular Biochemistry University of Kentucky Lexington Kentucky USA; ^5^ Department of Biomedical Engineering University of Kentucky Lexington Kentucky USA; ^6^ Department of Biology University of Kentucky Lexington Kentucky USA

**Keywords:** Alzheimer's disease, amyloid beta, circadian rhythms, cytokines, glia, knock‐in mouse model, MW151, nesting behavior, neuroinflammation, radial arm water maze, sleep

## Abstract

**INTRODUCTION:**

Sleep and circadian disturbances are early Alzheimer's disease (AD) features, yet mechanisms linking amyloid pathology, neuroinflammation, and sex differences remain unclear.

**METHODS:**

We longitudinally assessed sleep, circadian rhythms, and cognition in female and male hAPP^SAA^ knock‐in and control mice from 2 to 19 months using piezoelectric monitoring. Aged mice (15 months) received MW151, a glial cytokine inhibitor (2.5 mg/kg, every other day, 6 weeks).

**RESULTS:**

Only females exhibited midlife reductions in light‐phase sleep, increased rhythm fragmentation, and reduced rhythm stability, coinciding with selective reversal learning deficits, effects independent of amyloid or cytokine burden. MW151 increased light‐phase sleep and reduced cortical TNF‐α without altering amyloid beta (Aβ) accumulation.

**DISCUSSION:**

HAPP^SAA^ mice recapitulate female‐predominant non‐cognitive AD features, including sleep fragmentation and circadian instability preceding memory deficits. Sleep improved within weeks of MW151 treatment without Aβ reductions, implicating neuroinflammatory signaling as a rapid, modifiable driver of AD‐related sleep disruption.

## Highlights

- Female SAA mice develop midlife sleep and circadian deficits before memory loss.
- Sex differences in sleep disruption occur despite equivalent amyloid burden.
- Cortical TNF‐α is reduced by MW151 while glial markers remain unchanged.
- Glial cytokine modulation rescues sleep independent of amyloid clearance.

## 1 BACKGROUND

Sleep disturbances and circadian rhythm disruption are among the earliest and most consistent non‐cognitive symptoms of Alzheimer's disease (AD), often appearing years before measurable cognitive decline. Fragmented sleep and reduced sleep efficiency (time in sleep relative to time in bed) predict disease risk and correlate with greater amyloid and tau pathology.^1^, ^2^, ^3^, ^4^, ^5^, ^6^, ^7^ As pathology progresses, patients exhibit irregular rest‐activity rhythms characterized by increased nighttime awakenings, late‐day hyperactivity (“sundowning”), and blunted circadian rhythm amplitude.^8^, ^9^, ^10^ Actigraphy studies confirm that increased intradaily variability (IV) and diminished stability of circadian rhythms often precede AD diagnosis and that amyloid‐positive older adults show elevated late‐day activity.^11^, ^12^ Disrupted sleep may further accelerate disease progression by impairing glymphatic clearance, promoting amyloid beta (Aβ) accumulation, and sustaining neuroinflammation, thereby creating a bidirectional cycle between sleep disruption and pathology.^13^, ^14^, ^15^, ^16^, ^17^, ^18^

These sleep–wake alterations show pronounced sex differences, such that women with AD and females of AD‐related mouse models exhibit greater sleep fragmentation and circadian rhythm disruption than males despite similar amyloid burden.^2^, ^19^ In both humans and rodents, midlife represents a period of increased female vulnerability, coinciding with biological, hormonal, and neuroimmune changes that may alter circuits regulating sleep and cognition.^20^, ^21^, ^22^ These observations suggest that sex and aging interact to influence the onset and severity of sleep–wake dysregulation in AD. Yet, the mechanistic basis for these interactions remains poorly understood, an important gap this study aims to address.

Parallel alterations in sleep and circadian organization have been documented in AD mouse models, strengthening their translational relevance. For instance, tauopathy lines such as PS19 exhibit early sleep disruption, with females showing dark‐phase hyperarousal by 3 months and males by 6 months.^23^ Similarly, 5xFAD mice display fragmented sleep and shortened sleep–wake bouts by midlife and these alterations are more pronounced during the dark phase and in females.^24^ However, transgenic overexpression can accelerate pathology in ways that diverge from human disease by producing non‐physiological protein levels and off‐target effects.^25^, ^26^ Knock‐in (KI) strategies overcome these limitations by introducing human familial AD mutations into the endogenous amyloid precursor protein (APP) locus, preserving physiological expression. The hAPP^SAA^ KI incorporates the Swedish (KM670/671NL), Arctic (E693G), and Austrian (T714I) mutations and develops progressive amyloid deposition, gliosis, and transcriptional changes with age while avoiding the confounds of overexpression of APP.^27^ This model exhibits variable, task‐dependent cognitive phenotypes that emerge with aging, making it well suited for longitudinal assessment of sleep and cognitive changes.^28^, ^29^

To capture the temporal evolution of these phenotypes, we used a longitudinal design spanning from before pathology to late‐stage disease. Sleep and circadian activity were recorded starting at 2 months of age, a stage preceding detectable Aβ accumulation, to establish baseline physiology. Mice were then monitored for 19 months to capture changes associated with progressive Aβ, providing a molecular context for age‐related alterations in sleep and behavior.

Cytokines such as interleukin (IL)‐1β and tumor necrosis factor alpha (TNF‐α) can acutely promote sleep but, when chronically elevated, disrupt normal sleep–wake architecture and shift neuroimmune signaling from homeostatic to pathological modes.^30^, ^31^, ^32^ Chronic glial cytokine overproduction may therefore represent a mechanistic link between neuroinflammation and sleep disruption in AD. To directly test this, we treated aged SAA mice with MW151, a selective, brain‐penetrant small molecule that suppresses glia‐mediated cytokine overproduction without impairing baseline immune function.^33^ We combined non‐invasive home‐cage piezoelectric sleep monitoring with circadian analysis and assessed nest building (a proxy for daily‐living function) and hippocampal‐dependent cognition, including reversal learning (executive flexibility). Biochemical analyses of Aβ and cytokine/chemokine levels were performed to assess relationships between pathology, inflammation, and behavioral phenotypes.

We hypothesized that hAPP^SAA^ KI mice would exhibit reduced light‐phase sleep, greater rhythm fragmentation, and selective deficits in cognitive flexibility and that these abnormalities could be ameliorated by MW151 treatment. Together, these studies identified glial inflammation as a target to restore sleep and cognitive health in AD and suggest changes in sleep quality as a translatable biomarker of response to glia‐targeted anti‐inflammatory therapy.

## 2 METHODS

### 2.1 Animals

A total of 352 mice were included across five experimental cohorts. hAPP^SAA^ KI (SAA; JAX stock no. 034711) and hAPP^WT^ KI (wild type [WT]; JAX stock no. 033013) mice were bred in‐house at the University of Kentucky. Homozygous mice were used for all experiments. All procedures were approved by the University of Kentucky Institutional Animal Care and Use Committee (IACUC; Protocol: 2018‐3066) and conducted in accordance with ARRIVE guidelines. Mice were group‐housed (up to five per cage) under controlled temperature (22 ± 2°C) and humidity (40% to 60%), with food and water available ad libitum. Housing was maintained on a 12:12 h light/dark cycle (lights on at 07:00 [Zeitgeber time 0, ZT0], off at 19:00 [ZT12]).

### 2.2 Study design

Mice were tested across five independent cohorts, with both sexes represented. Testing began at 2 months of age to establish a pre‐pathology baseline, prior to the onset of Aβ accumulation, which typically begins around 4 months in this model.^27^ Longitudinal sleep and circadian assessments were conducted through 19 months to capture progressive changes across aging and advanced pathology. Learning and memory were evaluated in early adulthood (4 months) to assess whether early Aβ deposition impacts cognition. In parallel, biochemical analyses of Aβ and inflammatory markers (Meso Scale Discovery [MSD] cytokine/chemokine assays) were performed from 1 to 15 months, spanning the pre‐pathology stage through midlife and early advanced pathology.

In addition, a fifth cohort was used to evaluate the impact of pharmacological modulation of neuroinflammation on sleep and circadian rhythms. In this cohort, aged SAA mice (∼15 months) received the glial cytokine‐suppressing compound MW151 or vehicle for 6 weeks, with sleep recordings conducted longitudinally before and during treatment to determine whether attenuation of inflammatory signaling ameliorated age‐related sleep and circadian disruption.

Cohort 1 included female WT (*n =* 8) and SAA (*n =* 8) mice and male WT (*n =* 8, one excluded due to death) and SAA (n *=* 8, one excluded due to death) mice that were monitored longitudinally from 2 to 19 months of age. Sleep and circadian rhythms were assessed using piezoelectric home‐cage monitoring (Signal Solutions, Lexington, KY, USA) at 2, 4, 6, 8, 10, 15, 17, and 19 ± 0.3 months (mean age ± standard deviation [SD]) for seven consecutive days. Nesting behavior was evaluated at 6, 8, 10, 13, 15, 17, and 19 ± 0.3 months (mean age ± SD). When not being monitored, mice were group‐housed to reduce stress. Data from the 13‐month time point in Cohort 1 were excluded due to technical complications during sleep recording.

Cohort 2 consisted of younger mice (4.28 ± 0.3 months; mean age ± SD) tested in the eight‐arm radial arm water maze (RAWM) to assess hippocampus‐dependent spatial learning and memory. This cohort included female WT (*n =* 9) and SAA (*n =* 7) mice and male WT (*n =* 10) and SAA (*n =* 11) mice.

Cohort 3 consisted of older mice evaluated in RAWM acquisition, probe, and reversal learning, with nesting behavior assessed in parallel. This cohort included female WT ( n = 13) and SAA (*n =* 12) mice and male WT (n = 11) and SAA (*n =* 13) mice. Two SAA mice, one female (excluded due to microphthalmia in the right eye) and one male (excluded due to death), were omitted from RAWM testing. For nesting behavior, two males were excluded, one that died prior to RAWM testing and another that died before the final nesting assessment. For Cohort 3, RAWM acquisition occurred at 9.2 ± 0.4 months, nesting at 10.5 ± 0.4 months, RAWM probe and reversal at 11.0 ± 0.4 months, and the final nesting assessment at 13.3 ± 0.4 months (mean age ± SD). Also, Cohort 3 was tested in locomotor activity prior to RAWM acquisition (8.87 ± 0.42 months; mean age ± SD).

**RESEARCH IN CONTEXT**

1. **Systematic review**: We reviewed PubMed for studies linking amyloid pathology, neuroinflammation, sleep disruption, and sex differences in AD models and human cohorts. While sleep disturbances are recognized as early AD features, mechanistic studies examining sex‐specific vulnerability and glial contributions in KI models are limited.
2. **Interpretation**: Female hAPP^SAA^ KI mice develop midlife sleep fragmentation and circadian instability independent of amyloid or cytokine burden, suggesting heightened sensitivity to aging and glial signaling. MW151 improved rest‐phase sleep without altering Aβ, implicating neuroinflammatory pathways as rapid, modifiable drivers of sleep disruption.
3. **Future directions**: Studies should determine whether glial cytokine modulation prevents or delays cognitive decline longitudinally, identify the neural circuits linking cortical inflammation to sleep‐regulatory nuclei, and test whether these sex differences translate to human prodromal AD.

Cohort 4 included WT (*n =* 31 females, 34 males) and SAA (*n =* 34 females, 35 males) mice sacrificed at multiple ages spanning 1 to 16 months. Neocortical tissue was collected for biochemical analyses, including Aβ40/42 quantification and multiplex MSD assays for cytokines and chemokines, to characterize the progression of amyloid accumulation and neuroinflammation. A subset was used for immunohistochemical analysis (WT: 26 females and 27 males; SAA: 23 females, 26 males).

Cohort 5 (MW151 intervention study) consisted of SAA mice (*n =*  48 females and 52 males), aged 15.05 ± 0.74 months (mean age ± SD) at the start of the experiment and a lower number of WT mice. This cohort was designed to test whether pharmacological suppression of glia‐dependent cytokine overproduction could ameliorate sleep and circadian disturbances in aged SAA mice. Sleep–wake behavior was recorded using the piezoelectric home‐cage monitoring (Signal Solutions, Lexington, KY, USA) for 1 week prior to treatment to establish individual baselines. Mice were then randomly assigned to receive MW151 or vehicle for 6 weeks, with sleep recordings conducted during baseline, and at treatment weeks 3 and 5. When not being monitored, mice were group‐housed under standard 12:12 h light–dark conditions.

Locomotor activity and RAWM testing occurred during the light phase (08:00 to 16:00). Mice were acclimated to the testing room for at least 20 min and handled on three consecutive days prior to testing. All the experiments were performed by experimenters blinded to genotype and treatment condition. Group assignments were decoded only after data collection and initial quality control were complete. For longitudinal sleep studies, experimenters could not be blinded to age but remained blinded to genotype throughout data acquisition and analysis.

### 2.3 Circadian and sleep recordings

Daily rhythms in sleep–wake patterns were assessed using the PiezoSleep system (Signal Solutions, Lexington, KY, USA), a validated non‐invasive home‐cage monitoring platform for rodent sleep analysis.^34^, ^35^, ^36^ Mice were transferred to the testing room 1 week prior to recordings and then housed individually in cages equipped with piezoelectric floor sensors. Continuous sleep–wake monitoring was then conducted for seven consecutive days under a 12:12 light–dark cycle (lights on at 07:00, off at 19:00), with food and water available ad libitum, and to ensure acclimation, the first 2 days of recordings were excluded from analysis. At the end of the recording period, animals were returned to group housing.

The PiezoSleep system detects subtle pressure changes in the cage floor associated with movement. Sleep was defined as sustained immobility punctuated only by respiration signals, whereas wake was defined as periods of active movement. Sleep–wake data were processed using SleepStats software (version 2.181, Signal Solutions, Lexington, KY, USA). Primary sleep parameters included percent sleep during (1) the 12‐h light phase, (2) the 12‐h dark phase, and (3) the full 24‐h period. Movement data were transformed into continuous activity scores ranging from 0 (sleep) to 3 (near‐continuous motion). These data were exported into ClockLab software (version 6.1, Actimetrics) for circadian rhythm analysis. Cosinor analysis was applied to fit a sine wave to the activity profile, yielding amplitude (the peak‐to‐trough difference, reflecting rhythm robustness) and the Midline Estimating Statistic of Rhythm (MESOR), the modeled rhythm‐adjusted mean activity. In addition, non‐parametric circadian rhythm analysis (NPCRA) provided complementary measures: IV, indexing rhythm fragmentation, interdaily stability (IS), and indexing day‐to‐day consistency. All recordings and analyses were performed by experimenters blinded to genotype and sex. Sleep data from the 13‐month time point in Cohort 1 were excluded due to technical complications during acquisition.

### 2.4 Nesting behavior

Nesting behavior was assessed as an ethologically relevant measure of daily living activity. Mice were placed in clean standard cages for at least 30 min beginning at 15:00. Then, a single pressed cotton nestlet (5 × 5 cm; Ancare, Bellmore, NY, USA) was added to each cage. The following morning, approximately 16 h later, nest quality was scored by observers blinded to genotype and experimental conditions. Nest quality was scored on a semi‐quantitative five‐point scale, where 1 indicated an untouched nestlet, 2 a partially torn nestlet, 3 a mostly shredded nestlet without an identifiable nest site, 4 an identifiable but flat nest, and 5 a near‐complete crater‐ or dome‐like nest.^37^ In Cohort 1, nesting was assessed between day 1 and day 2 of sleep recordings, with scores from three blinded observers averaged for analysis. In Cohort 3, nesting was conducted in parallel with RAWM testing, with scores averaged from two blinded observers. After evaluation, mice were returned to their original group‐housed cages.

### 2.5 Radial arm water maze

Spatial learning and memory were tested in an eight‐arm radial arm water maze (RAWM) following the protocol previously described by our laboratory.^38^ The circular pool (121 cm in diameter, 75 cm deep) was filled with opaque water maintained at 22 ± 1°C. Black curtains surrounded the pool to block extraneous cues, and distal visual cues were affixed to the curtains and kept constant across testing. A hidden escape platform (8 cm in diameter) was submerged 1 cm below the surface at the end of one designated goal arm.

Mice completed 28 trials across four consecutive days, with seven trials per day. On days 1 and 2, the platform was visible (training), and on days 3 and 4, it was hidden but remained in the same goal arm (acquisition). Trials were divided into two blocks per day to minimize fatigue. If a mouse failed to locate the platform within 60 s, it was guided to the platform and allowed to remain there for 15 s. Mice exhibiting persistent floating or swimming impairments were excluded. Cohort 2 completed only the initial 4‐day RAWM protocol. Cohort 3 underwent the same training and acquisition phases, followed approximately 2 months later by a second testing phase that included 2 days of probe testing (days 5 and 6), during which the platform remained in the original arm, and 2 days of reversal learning (days 7 and 8), during which the platform was relocated to a new arm while cues remained constant. All probe and reversal trials used hidden platforms and the same staggered seven‐trial‐per‐day design. Behavioral data were collected and analyzed using EthoVision XT 11.0 (Noldus Information Technology).

### 2.6 Drug treatment

MW151, a small‐molecule inhibitor of glia‐mediated proinflammatory cytokine overproduction, was administered to SAA mice in Cohort 5. Mice received MW151 at a dose of 2.5 mg/kg via oral gavage every other day during the light phase (ZT3) for 6 weeks. The compound was dissolved in sterile saline immediately before administration. Control mice received an equivalent volume of vehicle (sterile saline) on the same schedule. MW151 is orally bioavailable, and the dose and route are consistent with Hu et al.^51^ Oral gavage was selected over intraperitoneal injection to minimize handling stress, which is particularly important given that sleep is the primary outcome measure. The selected dose and regimen were based on prior work demonstrating that MW151 effectively suppressed glial overproduction of IL‐1β and TNF‐α, attenuated neuroinflammation, and improved behavioral outcomes in models of neurodegeneration and brain injury, without altering basal cytokine levels or normal immune function.^33^, ^39^, ^40^ Sleep was recorded at baseline (prior to dosing) and during treatment weeks 3 and 5. Primary statistical comparisons were prespecified at week 5 and performed between vehicle‐ and MW151‐treated mice at that time point, when both groups underwent identical handling and gavage procedures. Baseline values were used only to center each animal on its own starting value and were not used for inferential comparisons.

### 2.7 Histology and biochemical analyses

Mice from Cohort 4 were deeply anesthetized with 5% isoflurane in 100% O_2_ and transcardially perfused with ice‐cold 1× phosphate buffered saline (PBS) for 5 min (ZT2‐3). Mice from Cohort 5 were euthanized with CO_2_ at ZT6. Brains were rapidly removed, and the left hemibrain was fixed in 4% paraformaldehyde (PFA) for 24 h, then transferred to 30% sucrose until sectioning for immunohistochemistry (IHC). The right neocortex and hippocampus were dissected, flash‐frozen in liquid nitrogen, and stored at −80°C for biochemical analyses.

Fixed brain hemispheres were coronally sectioned at 30 µm using a freezing microtome, and every 10th section between approximately 1.3 mm and −2.5 mm from bregma was collected for staining. Systematic sampling every 10th section yielded approximately eight sections per animal spanning 1.3 to −2.5 mm from bregma, which were immunostained and quantified for each marker. IHC was performed on free‐floating sections following previously established protocols.^33^, ^41^ The following primary antibodies were used: (1) mouse anti‐Aβ (clone 6E10, 1:3000; BioLegend, Catalog No.: 803007, RRID: AB_2564657) to detect Aβ, (2) rabbit anti‐glial fibrillary acidic protein (GFAP) (1:10,000; Dako, Catalog No.: Z0334, RRID: AB_10013382) for astrocytes, (3) rabbit anti‐IBA1 (1:10,000; Wako, Catalog No.: 019‐19741, RRID: AB_839504) for microglia, (4) rat anti‐CD‐45 (1:1000; BioLegend, Catalog No.: 103102, RRID: AB_312967) for microglia/macrophages and lymphocytes, and (5) rat anti‐Dectin‐1 (1:1000; InvivoGen, Catalog No.: mabg‐mdect, RRID: AB_2753143) for disease‐associated microglia. Slides were scanned using a Zeiss AxioScan Z.1 digital slide scanner, and quantitative image analysis was performed using HALO software (Indica Labs, Albuquerque, NM, USA). The neocortex and hippocampus were manually outlined, and the percent area covered by positive staining was quantified using the HALO Area Fraction algorithm. Values were normalized to total outlined area to account for regional size differences. Aβ plaque density (plaques/mm^2^) was quantified and further classified by size using the HALO Object Colocalization algorithm, applying predefined thresholds for small (10 to 99 µm^2^), medium (100 to 399 µm^2^), and large (≥400 µm^2^) plaques, following established methods.^41^, ^42^ All image processing and quantification were conducted in batch mode with identical parameters by an experimenter blinded to treatment group.

For biochemical analyses, frozen tissue was sequentially homogenized to obtain PBS‐, detergent‐, and formic acid (FA)‐soluble fractions, as previously described.^33^, ^41^ Briefly, homogenization was first performed in PBS containing 1 mM EDTA and protease inhibitors, followed by detergent extraction (T‐PER with Halt protease/phosphatase inhibitor cocktail, Thermo Fisher Scientific), and finally FA extraction (70% FA). Supernatants were collected after centrifugation at 12,000 × g for 20 min at 4°C (PBS and detergent fractions) or 100,000 × g for 60 min at 4°C (FA fractions).

Levels of Aβ_40_ and Aβ_42_ were quantified using MSD electrochemiluminescent Enzyme‐Linked Immunosorbent Assay (ELISA) kits according to the manufacturer's instructions. Total Aβ was quantified using human‐specific Aβ1‐16 antibody 42.5 (1 µg/well) and detected with biotinylated anti‐Aβ17‐24 (4G8, BioLegend 800701, RRID:AB_2728526). Cytokines (IL‐1β, TNF‐α, and IL‐10) were measured using MSD multiplex immunoassays. Protein concentrations were determined by bicinchoninic acid assay, and analyte values were normalized to total protein concentration.

### 2.8 Statistical analysis

Data are presented as mean ± standard error of the mean (SEM) for continuous outcomes and median (interquartile range) for ordinal outcomes. Sleep and circadian data were analyzed in SPSS version 29 (IBM, Armonk, NY, USA), while RAWM, nesting, and Cohort 5 (MW151 treatment) sleep data were analyzed in JMP 18 Student Edition (SAS Institute, Cary, NC, USA). Graphs were generated in GraphPad Prism version 10.6.0 (GraphPad Software, San Diego, CA, USA). Sleep and circadian variables were analyzed using repeated‐measures ANOVA, with Greenhouse–Geisser correction applied when assumptions of sphericity were violated. Circadian parameters (amplitude, MESOR, IS, IV) were evaluated within the same framework. Normality and variance assumptions were tested with Shapiro–Wilk and Levene's tests, and data were transformed when necessary. Nesting scores were compared between genotypes using Wilcoxon rank‐sum tests, and effect sizes were reported as Hodges–Lehmann estimates with 95% confidence intervals (CIs). RAWM acquisition and retention were analyzed with repeated‐measures ANOVA (Day X Genotype), while reversal learning was analyzed with repeated‐measures ANOVA and, when appropriate, unpaired *t*‐tests. Area under the curve (AUC) of cumulative errors was compared between WT and SAA mice within each sex using independent *t*‐tests. Effect sizes (partial η^2^) were interpreted as small (0.01), medium (0.06), or large (≥0.14) following Cohen.^43^ For the MW151 treatment cohort (Cohort 5), post‐treatment values were normalized as percent change from baseline, calculated by subtracting each mouse's baseline value from its week 5 value, dividing by baseline, and multiplying by 100. This normalization centers each animal on its own starting value to reduce interanimal variability. Statistical inference was performed between treatment groups at week 5 only. Primary sleep inferences were based on the percent change scale, while some panels display raw week 5 values for visualization. Week 5 was prespecified as the primary time point, with light‐phase sleep and MESOR designated as primary endpoints; dark‐phase sleep, total sleep, IV, and amplitude as secondary endpoints; and cytokines as secondary biochemical measures. Forest plots summarize week 5 treatment effects as least‐squares mean differences (MW151 minus vehicle), with 95% CIs derived from the ANOVA model. A two‐way ANOVA was used with fixed factors Treatment (vehicle, MW151), Sex (female, male), and their interaction (Treatment × Sex), using sums of squares. When the interaction was non‐significant, inference for treatment effects was based on the main effect collapsed across sex. Where appropriate, simple least‐squares mean contrasts were reported within sex. Effect sizes are reported as partial η^2^ for ANOVA terms and least‐squares mean differences with 95% CIs for contrasts. Statistical significance was set at *p <* 0.05.

## 3 RESULTS

### 3.1 hAPP^SAA^ mutations reduce light‐phase sleep in female mice beginning in midlife

**FIGURE 1:**
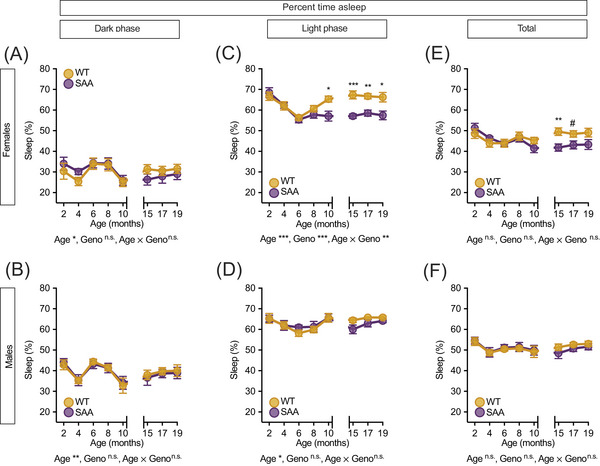
hAPP^SAA^ KI female mice show reduced light‐phase sleep beginning at midlife. Sleep–wake patterns were assessed longitudinally in wild‐type (WT) and SAA mice using piezoelectric home‐cage sensors from 2 to 19 months of age (A–F). (A and B) Dark‐phase sleep (active period) showed similar age‐related variation between genotypes in both female (A) and male (B) mice. (C and D) Light‐phase sleep (rest period) was progressively reduced in SAA females (C) beginning at 10 months and persisting with age, with significant genotype differences detected at 10, 15, 17, and 19 months. In males (D), age‐dependent changes were evident, but no genotype differences were observed at any time point. (E and F) Total sleep was reduced in SAA females (E) at 15 months and remained marginally reduced at 17 months (^#^ *p =* 0.0507), whereas total sleep in males (F) did not differ between genotypes. Data are presented as mean ± SEM. Asterisks denote statistical differences (*p < 0.05, **p < 0.01, ***p < 0.001). Statistical outcomes from repeated‐measures ANOVA for main effects of age, genotype, and age × genotype interactions are reported below each graph. Females, WT *n =* 8, SAA *n =* 8; males, WT *p=n*  7, SAA *=* 7. Analysis by time point is shown in Table S1.

Piezoelectric sleep recordings revealed age‐related changes in percent sleep across both sexes, but genotype effects were confined to females. During the dark phase (corresponding to the most active phase), females showed a significant age effect (*F*[3.8, 52.6] = 3.074, *p =* 0.026), but no genotype effect or interaction (Figure 1A). Males similarly showed an age effect (*F*[7, 98] = 6.493, *p =* 0.006) without genotype differences (Figure 1B). During the light phase (corresponding to the most inactive phase), genotype (*F*[1, 14] = 28.75, *p <* 0.001), age (*F*[7, 98] = 6.60, *p <* 0.001), and an age × genotype interaction (*F*[7, 98] = 3.33, *p =* 0.012) were seen in the female mice. The reduction in light‐phase sleep first emerged at midlife (10 months: *p =* 0.011) and persisted with aging, with SAA females sleeping significantly less than WT controls at 15 (*p =* 0.0004), 17 (*p =* 0.0012), and 19 months (*p =* 0.014) (Figure 1C). By 19 months, SAA females slept 8.8% less than WT counterparts. In males, only an age effect was detected (*F*[7, 98] = 4.05, *p =* 0.018); both genotypes showed similar light‐phase sleep across all ages, with no significant genotype effect or interaction (all *p >* 0.15) (Figure 1D). Across the 24‐h cycle (total sleep), females exhibited an effect of age (*F*[4.59, 64.23] = 2.42, *p =* 0.050) (Figure 1E), while no significant changes were seen in males (Figure 1F). Together, these results indicate that in SAA mice, sleep disruption occurs in females, emerging around midlife as reduced light‐phase sleep. In contrast to females, SAA males maintain stable sleep patterns across aging, and these patterns did not differ significantly from those of WT mice. Comprehensive statistical outputs for percent sleep across ages and phases are provided in Table S1, and complementary analyses of sleep–wake transitions are presented in Figure S1 and Table S2.

### 3.2 hAPP^SAA^ mutations alter circadian rhythm robustness and stability in females but not males

**FIGURE 2:**
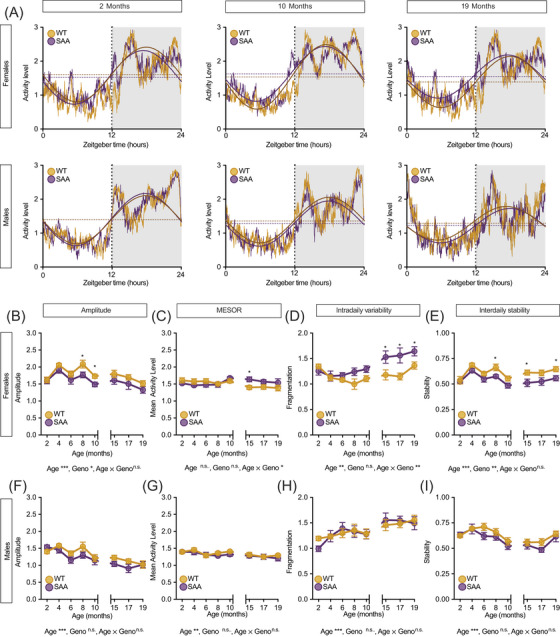
hAPP^SAA^ mutations increase sleep fragmentation and decrease circadian rhythm stability in aging female mice. Representative 24‐h activity waveforms (A) and group‐averaged circadian parameters (B–I) show age‐related changes in both sexes, with genotype effects restricted to females. In females (B–E), SAA mice exhibited lower amplitude at 8 and 10 months, transiently higher MESOR at 15 months, and increased intradaily variability together with reduced interdaily stability beginning in midlife. Males (F–I) showed comparable age‐related reductions in rhythm amplitude and stability but no genotype‐dependent differences. Data are presented as mean ± SEM. Asterisks denote statistical differences (*p < 0.05, **p < 0.01, ***p < 0.001). Statistical outcomes from repeated‐measures ANOVA for main effects of age, genotype, and age × genotype interactions are reported below each graph. Females, wild type (WT) *=* 8, SAA *n =* 8; males, WT *=* 7, SAA *n =n* 7. Analysis by time point is shown in Table S2.

To determine whether these reductions in sleep in SAA females were accompanied by changes in circadian organization, we next examined rhythm fragmentation, amplitude, and IS across the same period. Analysis of circadian parameters revealed progressive, sex‐dependent changes in rhythm robustness and stability, with representative 24‐h activity profiles shown in Figure 2A and Figure S2. In females (Figure 2B), amplitude declined significantly with age (*F*[7, 98] = 8.79, *p =* 0.002) and showed a main effect of genotype (*F*[1, 14] = 5.78, *p =* 0.031). SAA females exhibited lower amplitude than WT at 8 months (*p =* 0.0447) and 10 months (*p =* 0.0235). MESOR, representing the mean 24‐h activity level, did not differ significantly by genotype or age overall (Figure 2C). IV, an index of rhythm fragmentation, increased with age (*F*[7, 98] = 6.53, *p =* 0.001) and showed a significant age × genotype interaction (*F*[7, 98] = 2.42, *p =* 0.014). SAA females displayed greater IV than WT beginning at 15 months (*p =* 0.0437) and persisting through 17 (*p =* 0.0229) and 19 months (*p =* 0.0297), indicating progressively more frequent transitions between rest and activity states (Figure 2D). IS, reflecting day‐to‐day rhythm coherence, declined with age (*F*[7, 98] = 5.98, *p <* 0.001) and showed a robust genotype effect (*F*[1, 14] = 18.06, *p =* 0.001). SAA females exhibited reduced IS compared with WT at 8 (*p =* 0.0416), 15 (*p =* 0.0416), and 19 months (*p =* 0.0331), indicating diminished circadian regularity and greater variability between days (Figure 2E). In males, amplitude (*F*[3.39, 40.72] = 16.27, *p <* 0.001) (Figure 2F), MESOR (*F*[7, 84] = 3.39, *p =* 0.003) (Figure 2G), IV (*F*[7, 84] = 8.32, *p <* 0.001) (Figure 2H), and IS (*F*[7, 84] = 11.18, *p <* 0.001) (Figure 2I) each showed significant age effects, consistent with an overall age‐related decline in rhythm robustness. However, no genotype differences or interactions were detected in the males for any parameter across ages (all *p >* 0.05). Thus, the hAPP^SAA^ mutations selectively exacerbate rhythm fragmentation and instability in females, suggesting an early, sex‐dependent breakdown of circadian control mechanisms. Full circadian parameter values are reported in Table S3.

### 3.3 Nesting performance deteriorates with age and genotype in hAPP^SAA^ knock‐in mice

**FIGURE 3:**
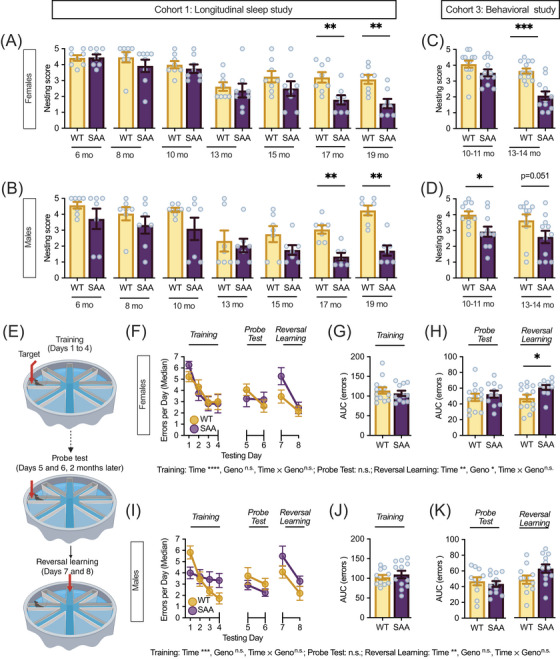
Age‐dependent nesting decline and sex‐specific reversal learning deficits in hAPP^SAA^ KI mice. Group‐averaged nest quality scores (A–D) show progressive, sex‐influenced impairments in nesting performance. In the longitudinal cohort, nest quality declined with age in both sexes, with genotype differences emerging at later time points (A and B). In the cross‐sectional cohort, impairments appeared earlier, with female SAA mice showing reduced nest quality at midlife and males displaying early‐onset deficits (C and D). Together, these findings indicate that SAA mutations lead to age‐ and sex‐dependent reductions in daily‐living function, consistent with progressive decline in ethological measures of functional capacity. Cohort 1 consisted of mice evaluated longitudinally at seven time points (6 to 19 months) as part of a sleep study (A, females: eight WT/eight SAA; B, males: seven WT/seven SAA). Cohort 3 consisted of mice evaluated at two time points during a separate behavioral study (C, females: 13 WT/12 SAA; D, males: 11 WT/11 SAA). These cohorts represent independent experiments and are not intended for direct statistical comparison with one another. (E) Experimental design for the 8‐day radial arm water maze paradigm. Mice were trained to locate a hidden escape platform during the acquisition phase (Days 1–4), tested 2 months later for memory retention (probe; Days 5–6), and subsequently tested for cognitive flexibility in a reversal phase with the platform relocated (Days 7–8). (F–H) Female performance: both WT and SAA mice successfully acquired the task across training days, as shown by a decrease in errors (F) and comparable cumulative errors (area under the curve [AUC]) during acquisition (G) and probe phases (H). During reversal learning, SAA females committed more errors than wild‐type (WT) controls, reflected in elevated AUC values (H). (I–K) Male performance: WT and SAA males displayed similar learning curves during acquisition (J) and probe performance (K). A trend toward increased errors during reversal learning was observed in SAA males but did not reach significance (K). Statistical outcomes from repeated‐measures ANOVA for main effects of day, genotype, and day × genotype interactions are reported below each graph. Females: WT (*=* 13) and SAA (n *=* 11); males: WT (*n* = 11) and SAA (*n* = 12) mice. Each dot represents an individual animal. Data are presented as mean ± SEM. Asterisks denote statistical differences (*p < 0.05, **p < 0.01, ***p < 0.001, ****p < 0.0001).

Nesting behavior was evaluated as an ethological measure of activities of daily living to assess functional decline associated with motor coordination, motivation, and executive function, and reductions in nest quality are widely seen in neurodegenerative disease models.^37^, ^40^ In the longitudinal sleep study cohort (Cohort 1), nesting performance declined progressively with age (linear contrast: *F*[1,182] = 80.72, *p <* 0.0001) in both sexes (Figure 3A: females; 3B: males), with deficits seen by 17 months in female (U = 93, Z = 2.60, *p =* 0.009) and male (U = 75, Z = 2.84, *p =* 0.0045) SAA mice, that persisted at 19 months old (female | U = 94.5, Z = 2.75, *p =* 0.006; male | U = 75.5, Z = 2.90, *p =* 0.0037). In the independent behavioral study cohort (Cohort 3), the nesting deficit in female and male SAA mice was replicated, and with the larger sample size, deficits were seen at 13 to 14 months old in the females (U = 93, Z = −3.41, *p =* 0.0006; Figure 3C) and 10 to 11 months old in the males (U = 163, Z = 2.39, *p =* 0.017; Figure 3D).

To formally test for sex differences across sleep, circadian, and nesting endpoints, we performed full factorial analyses including genotype, age in months, and sex (Table S4). These analyses revealed significant main effects of sex for all sleep and circadian endpoints (all *p <* 0.05), but not for nesting, confirming that males and females differ in baseline values. Significant genotype × sex interactions were observed for light‐phase sleep (*F*[1, 208] = 10.29, *p =* 0.002) and IV (*F*[1, 208] = 8.98, *p =* 0.003). For other endpoints, the genotype effect was similar across sexes despite differences in baseline values.

### 3.4 hAPP^SAA^ mice show intact learning but female‐biased reversal deficits in RAWM

We assessed cognitive performance at a younger age, when plaque deposition is beginning (4.28 ± 0.3 months; mean age ± SD; Figure S3), and at an older age with established plaque pathology (9.2 ± 0.4 months) using RAWM (Figure 3).^27^ At both ages the WT and SAA mice acquired the task, and no genotype differences were detected during acquisition. In the older cohort, we assessed long‐term retention 2 months later in a 2‐day probe, and performance did not differ significantly by genotype (Figure 3). We then evaluated cognitive flexibility with reversal learning on days 7 and 8. In females, SAA mice made more errors than WT (*t*[20.87] = −2.52, *p =* 0.021). In males, the genotype effect was not significant (*t*[20.93] = −1.85, *p =* 0.078) (Figure 3). Open‐field testing did not reveal genotype‐related locomotor deficits (Figure S4), indicating that the reversal effect is not attributable to altered activity.

### 3.5 Amyloid accumulation and neuroinflammatory responses increase with age in hAPP^SAA^ mice

**FIGURE 4:**
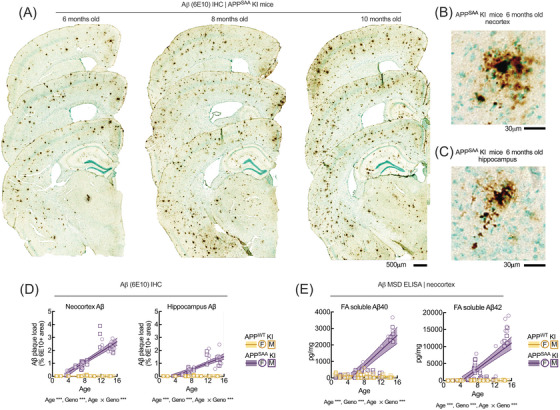
Progressive Aβ accumulation in neocortex and hippocampus of hAPP^SAA^ knock‐in (KI) mice. (A) Representative 6E10‐immunostained coronal sections show age‐dependent amyloid beta (Aβ) deposition in the neocortex and hippocampus of SAA mice, increasing markedly between 6 and 10 months of age. (B and C) Higher‐magnification images illustrate dense, compact plaques in the neocortex (B) and hippocampus (C) at 6 months. (D) Quantification of Aβ plaque burden revealed significant main effects of age and genotype, as well as an age × genotype interaction in both regions, reflecting accelerated Aβ accumulation in SAA mice. (E) Meso Scale Discovery (MSD) Enzyme‐Linked Immunosorbent Assay quantification of formic acid–soluble Aβ40 and Aβ42 in the neocortex confirmed these histological findings, with robust effects of age, genotype, and their interaction. Lines represent mean ± SEM with shaded 95% confidence intervals. Statistical outcomes from two‐way ANOVA (age, genotype, age × genotype) are shown below each panel. Asterisks denote statistical differences (***p < 0.001). Each point denotes an individual animal, circles represent females, and squares represent males. Statistical outcomes for age, genotype, and the interaction between age and genotype are reported in Table S5. Females: immunohistochemical/MSD wild type (WT) *=* 26/31 and SAA *n =* 23/34; males: WT *n =* 27/34 and SAA *n =* 26/35 mice.

**FIGURE 5:**
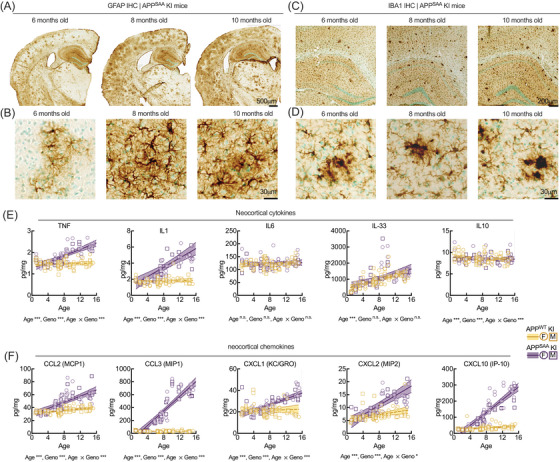
Age‐dependent astrogliosis, microgliosis, and cytokine/chemokine upregulation in hAPP^SAA^ KI mice. (A and B) Representative glial fibrillary acidic protein‐immunostained coronal sections show progressive astrogliosis in the neocortex and hippocampus of SAA mice from 6 to 10 months of age. (C and D) IBA1 immunostaining demonstrates parallel microglial activation and clustering around amyloid plaques in the same regions. (E and F) Quantification of neocortical cytokines and chemokines revealed robust age‐related increases in tumor necrosis factor alpha (TNF‐α), interleukin (IL)‐1β, IL‐33, CCL2, CCL3, CXCL1, CXCL2, and CXCL10, with the strong main effects of genotype and significant age × genotype interactions (see Table S3). In contrast, IL‐6 and IL‐10 showed no genotype‐dependent changes. Lines represent mean ± SEM with shaded 95% CIs. Statistical outcomes from two‐way ANOVA (age, genotype, age × genotype) are shown below each panel. Asterisks denote statistical differences (*p < 0.05, ***p < 0.001). Each point denotes an individual animal, circles represent females, and squares represent males. Statistical outcomes for age, genotype, and interaction effects are detailed in Table S5. Females: WT *n =* 31 and SAA *n =* 34; males: WT *n =* 34 and SAA *n =n* 34 mice.

Immunohistochemical and biochemical analyses revealed progressive Aβ deposition and inflammatory activation in SAA mice, with strong effects of both genotype and age (Figures 4 and 5; Table S5). Representative 6E10‐stained sections (Figure 4A–C) showed increasing Aβ plaque burden in the neocortex and hippocampus beginning around 6 months and intensifying through 10 months in SAA mice. hAPP^WT^ KI mice carrying the humanized Aβ1‐42 region did not develop detectable plaque pathology across the ages examined. Quantification (Figure 4D) confirmed large effects of genotype (neocortex: *F*[1, 102] = 93.59, *p <* 0.001, η^2^ = 0.48; hippocampus: *F*[1, 101] = 140.26, *p <* 0.001, η^2^ = 0.70) and age (neocortex: *F*[1, 102] = 130.38, *p <* 0.001, η^2^ = 0.56; hippocampus: *F*[1, 101] = 320.88, *p <* 0.001, η^2^ = 0.84), with strong age × genotype interactions in both regions (neocortex: *F*[1, 102] = 92.47, *p <* 0.001, η^2^ = 0.48; hippocampus: *F*[1, 101] = 139.65, *p <* 0.001, η^2^ = 0.70). ELISA quantification of FA‐extractable Aβ_40_ and Aβ_42_ (Figure 4E) confirmed these findings, showing significant main effects of genotype (*F*[1, 129] = 24.35 [Aβ_40_], 55.10 [Aβ_42_], *p <* 0.001) and age (*F*[1, 129) = 54.15(Aβ_40_), 73.57 (Aβ_42_), *p <* 0.001), with large age × genotype interactions (F(1, 129] = 59.98 [Aβ_40_], 73.24 [Aβ_42_], *p <* 0.001). Sex was included as a factor in all Aβ analyses, and no significant main effects of sex or interactions with sex were detected. Given the well‐known sex differences in amyloid pathology reported in transgenic APP models,^44^ we performed an exploratory analysis stratifying the data by sex (Figure 5). This analysis was not supported by significant sex effects but was conducted to allow direct visualization of the data. Both sexes showed similar patterns of age‐dependent Aβ accumulation with comparable age × genotype interactions, supporting the statistical results demonstrating that while sleep was sexually dimorphic, Aβ loads were not.

Consistent with amyloid pathology, SAA mice exhibited pronounced astroglial and microglial activation (Figure 5A–D). GFAP and IBA1 immunostaining revealed hypertrophic, densely stained glia in 6‐month‐old mice with visually increasing density at 8 and 10 months, indicating reactive gliosis. Multiplex cytokine analyses of neocortical tissue (Figure 5E,F) demonstrated broad inflammatory activation. TNF‐α and IL‐1β showed large effects of genotype (*F*[1, 129] = 66.38 and 121.95, both *p <* 0.001) and age (*F*[1, 129] = 35.89 and 55.69, both *p <* 0.001), with significant age × genotype interactions (*F*[1, 129] = 27.66 and 33.52, both *p <* 0.001). In contrast, IL‐6, IL‐33, and IL‐10 did not differ significantly across genotypes (*p >* 0.20). Among chemokines, CCL2, CCL3, CXCL1, CXCL2, and CXCL10 all exhibited robust effects of genotype (*F*[1, 129] = 31.99 to 456.74, all *p <* 0.001) and age (*F*[1, 129] = 16.64 to 110.36, all *p <* 0.001), with notable age × genotype interactions for CCL3 (*F*[1, 129] = 40.99, *p <* 0.001) and CXCL2 (*F*[1,129] = 6.71, *p =* 0.011). Sex was included as a factor in all cytokine and chemokine analyses. Of the analytes measured, only CXCL1 and CXCL2 showed significant sex‐related effects, with CXCL1 showing an age × sex interaction (*p =* 0.007) and CXCL2 showing a genotype × age × sex interaction (*p =* 0.014). To visualize these effects, data were stratified by sex in Figure S6. For CXCL1 and CXCL2, males showed stronger age × genotype interactions than females, whereas other cytokines and chemokines showed similar patterns in both sexes.

### 3.6 MW151 improves sleep and reduces inflammatory markers

Given these robust age‐dependent increases in amyloid pathology, gliosis, and cytokine overproduction, we next sought to determine whether selective attenuation of glia‐mediated cytokine signaling could mitigate these pathological and behavioral abnormalities. While the sleep phenotype was more pronounced in female SAA mice (Figures 1 and 2), elevated cytokines were observed in both sexes (Figure 5), suggesting that cytokine modulation could influence sleep outcomes within the SAA cohort, although the baseline sleep phenotype was more pronounced in females. To test this, a fifth cohort of 15‐month‐old SAA mice of both sexes received 6 weeks of treatment with MW151, a brain‐penetrant, small‐molecule modulator that normalizes cytokine output without broadly suppressing immune function.^33^, ^39^, ^40^, ^45^, ^46^, ^47^, ^48^, ^49^, ^50^, ^51^ Sleep was monitored longitudinally to evaluate whether reducing inflammatory tone (i.e., chronic, low‐grade cytokine milieu) could improve circadian rhythm stability and sleep integrity, followed by biochemical and histological analyses to assess MW151's impact on cortical cytokine expression and amyloid burden, and whether such effects would differ by sex.

While cytokines such as IL‐1β and TNF‐α are well known to acutely induce sleep, serving as endogenous sleep‐promoting signals, chronically elevated levels paradoxically disrupt normal sleep–wake architecture.^31^, ^32^, ^52^, ^53^, ^54^ The dose and duration of cytokine signaling may determine whether these molecules serve as beneficial homeostatic regulators or pathological disruptors of sleep.^55^, ^56^, ^57^ To mechanistically test whether glial cytokine overproduction was associated with the sleep and circadian abnormalities observed in SAA mice, we treated animals with MW151, a brain‐penetrant small molecule designed to selectively target glia‐dependent cytokine overproduction while maintaining physiological glial functions.^39^, ^40^, ^45^ We started treatment at ∼15 months, when they were treated by oral gavage with MW151 (2.5 mg/kg, every other day) or saline for 6 weeks.

**FIGURE 6:**
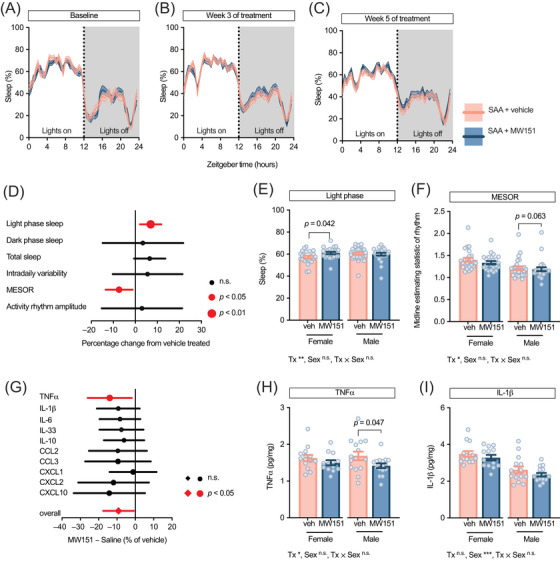
MW151 suppresses tumor necrosis factor alpha (TNF‐α) and reduces fragmented sleep in the hAPP^SAA^ knock‐in (KI) mice. At baseline, vehicle‐ and MW151‐assigned SAA mice were indistinguishable across the 24‐h profile (A). After 3 weeks of dosing, group means remained closely aligned (B). By week 5, MW151‐treated mice showed a visible elevation of sleep during the light phase, the normal rest period, relative to vehicle (C). Panel C displays mean 24‐h activity profiles to illustrate circadian organization; statistical comparisons of treatment effects are shown in panels (D) to (F). A forest plot summarizes the effects of MW151 on sleep and circadian parameters at the 5‐week time point (D). Light‐phase sleep increased with treatment, whereas MESOR decreased. Values are baseline‐corrected () and displayed as least‐squares mean differences for MW151 minus vehicle with 95% CIs. This was done to center each animal on its own starting value. Light‐phase sleep (E) and Midline Estimating Statistic of Rhythm (F) at week 5 show group summaries with individual animal distributions. These panels display raw week 5 values; all statistical tests were performed on baseline‐corrected outcomes shown in the forest plot (D). At week 6 (1 week after sleep recordings), a multiplex assay of PBS‐soluble neocortical homogenates showed a modest downward pattern across inflammatory analytes, with the “overall” value representing the mean of all measured cytokines and chemokines expressed as a percentage of vehicle (G). TNF‐α, the prespecified cytokine endpoint, was reduced by MW151, consistent with engagement of glia‐dependent cytokine output (H), whereas interleukin (IL)‐1β varied by sex but did not differ by treatment (I). In (A) to (C), lines show mean ± SEM across 24‐h Zeitgeber time. In (D) and (G), forest plots display mean differences with 95% confidence intervals. In (E), (F), (H), and (I), data are shown separated by sex to clearly demonstrate effects of sex as a biological variable; bars show mean ± SEM, with individual animals represented as circles. Main effects from two‐way ANOVA (treatment [Tx], sex, treatment by sex) are shown at the bottom of each panel; *p* values displayed on graphs (E, F, H, I; *p < 0.05, **p < 0.01, ***p < 0.001) represent post hoc contrasts comparing vehicle versus MW151 within each sex. (A–F) Veh/MW151 females:  n = 25/23 and males: *n =* 27/25; (G–I) Veh/MW151 females: *n =* 14/13 and males: *n =*  15/16 mice.

At baseline, prior to treatment, the two SAA groups were indistinguishable. The 24‐h sleep profiles of vehicle‐ and MW151‐assigned mice showed no differences in light‐phase sleep, dark‐phase sleep, total sleep, IV, MESOR, or activity rhythm amplitude (Figure 6A). After 3 weeks of MW151 administration, the sleep and circadian parameters remained largely unchanged between groups (Figure 6B). During the fifth week, MW151‐treated mice spent consistently more time asleep during the light phase (their natural rest period) compared to vehicle controls (Figure 6C–F). Baseline‐corrected values at the 5‐week time point showed a large effect of treatment (partial *η* ^2^ = 0.102) on light‐phase sleep (*F*[1, 66] = 8.03, *p =* 0.006), with no sex or treatment × sex interaction. Dark‐phase sleep, IV, and activity rhythm amplitude showed no significant treatment differences. The midline estimating statistic of the 24‐h rest–activity rhythm (MESOR) was also reduced with MW151 (*F*[1, 66] = 5.02, *p =* 0.028, partial *η* ^2^ = 0.081), indicating a downward shift in baseline activity consistent with more consolidated rest during the light phase while leaving rhythm amplitude unchanged. Total 24‐h sleep exhibited a medium effect of treatment (partial *η* ^2^ = 0.048), but this was not statistically significant (*F*[1, 66] = 3.33, *p =* 0.072). As seen in the time‐course data (Figure 1), there was a large (partial *η* ^2^ = 0.064) main effect of sex (*F*[1, 66] = 4.49, *p =* 0.03) on overall sleep; however, there were no treatment × sex interactions for any sleep or circadian endpoint (Table S6). Overall, MW151‐treated SAA mice exhibited increased light‐phase sleep relative to vehicle‐treated SAA mice. After 5 weeks, MW151 preferentially redistributed sleep toward the light phase and reduced baseline locomotor activity without altering amplitude or fragmentation. Although the absolute increase in light‐phase sleep was approximately 5%, the effect size was large (partial *η* ^2^ = 0.102). Moreover, this pattern is consistent with reduced daytime arousal tone (i.e., less cytokine‐driven wakefulness) rather than a weaker circadian pacemaker, suggesting stabilization of rest–activity patterns rather than a global sedative effect. The increased daytime sleep was also associated with the time of MW151 administration, that is, in the early light phase.

At week 6 of treatment, we measured a panel of cytokines and chemokines in PBS homogenates of neocortical tissue using multiplex assays. As shown in the forest plot (Figure 6G), MW151 produced an overall downward shift in inflammatory markers. Based on previous studies demonstrating that MW151 most robustly suppresses TNF‐α and IL‐1β while having minimal effects on chemokines, we focused our primary analysis on these inflammatory cytokines. TNF‐α was reduced by MW151 (*F*[1, 54] = 5.38, *p =* 0.024, partial η^2^ = 0.091) with no treatment × sex interaction (*p =* 0.423). Within‐sex post hoc tests were consistent with the main effect and suggested a larger reduction in males (males: *p =* 0.047; females: *p =* 0.242; Figure 6H). In contrast, IL‐1β showed no main effect of treatment (*p =* 0.118) or treatment × sex interaction (*p =* 0.823) but exhibited a strong sex effect with males producing substantially less IL‐1β than females (*F*[1, 54] = 37.56, *p <* 0.001, partial η^2^ = 0.410; Figure 6I). Other measured cytokines and chemokines (IL‐10, IL‐6, IL‐33, CCL2, CCL3, CXCL1, CXCL2, CXCL10) did not show treatment effects at week 6. These data indicate pharmacodynamic engagement, with selective reduction of cortical TNF‐α without broad chemokine suppression. The absence of an IL‐1β effect at this dose/time is compatible with higher basal IL‐1β levels in SAA mice or lower in vivo sensitivity of IL‐1β to MW151 under these conditions.

**FIGURE 7:**
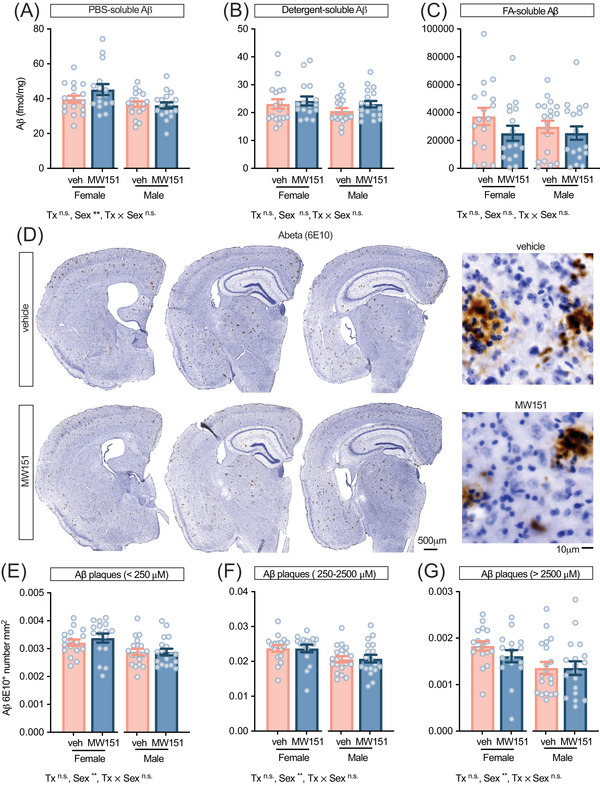
MW151 does not change amyloid beta (Aβ) levels or plaque burden. Aβ in (A) PBS‐, (B) detergent‐, and (C) FA‐soluble cortical fractions (fmol/mg) showed no effect of treatment. (D) Representative 6E10 immunostaining in neocortex (left: whole‐section montages; right: high‐magnification insets). (E–G) Neocortical plaque counts per square millimeter binned by area (<250 µm^2^, 250 to 2500 µm^2^, > 2500 µm^2^) using HALO‐AI classifier found no effect of treatment or treatment by sex interaction. Main effects from two‐way ANOVA (treatment [Tx], sex, treatment by sex; *p* value: **p < 0.01) are shown at the bottom of each panel. Points are individual hAPP^SAA^ knock‐in mice, bars: mean ± SEM. Veh/MW151 females: *n =*  17/16 and males: *n =* 19/18 mice.

In agreement with prior reports,^33^ MW151 did not alter amyloid measures in SAA mice (Figure 7A–G; Table S7). Aβ concentrations in PBS‐soluble, detergent‐soluble, and FA‐soluble fractions showed no treatment effect and no treatment × sex interaction (Figure 7A–C). Quantitative digital neuropathology of Aβ (6E10) IHC (Figure 7D) also showed no treatment effect on plaque number per square millimeter across size bins in the neocortex (Figure 7E–G) or hippocampus (Table S7).

**FIGURE 8:**
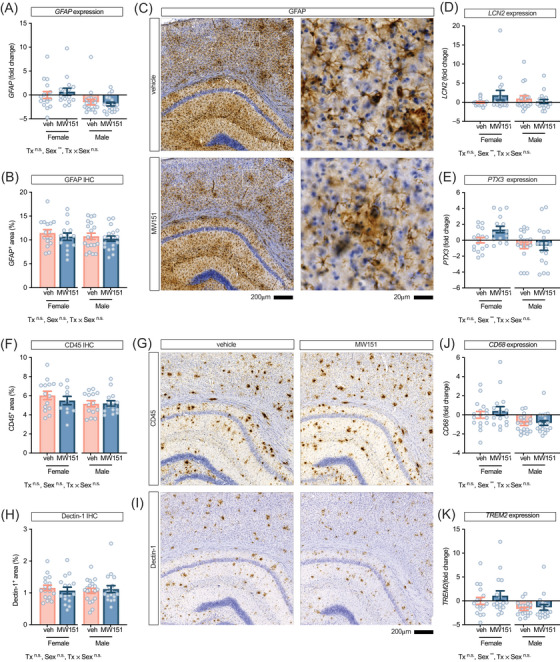
MW151 does not alter glial activation markers in neocortex. (A) Glial fibrillary acidic protein (GFAP) gene expression and (B) GFAP immunoreactivity showed no treatment effects. (C) Representative GFAP immunostaining (left: low magnification; right: high magnification) in hippocampus and neocortex from vehicle‐ and MW151‐treated mice. (D) *LCN2* and (E) *PTX3* gene expression were unchanged by treatment. (F) CD45 immunoreactivity quantification and (G) representative images from neocortex. (H) Dectin‐1 immunoreactivity and (I) representative images from neocortex. (J) *CD68* and (K) *TREM2* gene expression showed no treatment effects. Gene expression data (A, D, E, J, K) are fold change relative to vehicle; immunohistochemistry data (B, C, F, G, H, I) are percent area. Gene expression data are fold change relative to vehicle (both sexes combined). Immunohistochemistry data are percent area. Main effects from two‐way ANOVA (treatment [Tx], sex, treatment by sex; *p* value: **p < 0.01) are shown at the bottom of each panel. Points are individual hAPP^SAA^ knock‐in mice, bars: mean ± SEM. (A, D–K) Veh/MW151 females: *n =* 17/16 and males: *n =* 19/18. (B–H) Veh/MW151 females: *n =* 14 to 17/11 to 16 and males: *n =*14 to 19/15 to 18 mice.

To assess whether MW151's effects on TNF‐α production were accompanied by broader changes in glial activation states, we measured gene expression and protein levels of astrocyte and microglia markers in neocortex (Figure 8A–K). GFAP mRNA (Figure 8A) and GFAP immunoreactive area (Figure 8B–C) were not significantly changed by MW151. *GFAP* mRNA did show a main effect of sex (*F*[1, 66] = 11.3, *p =* 0.001, partial η^2^ = 0.146). *LCN2* mRNA (lipocalin‐2; astrocyte‐derived acute‐phase protein) was not significantly altered by treatment or sex (Figure 8D). *PTX3* mRNA (long pentraxin; glia‐secreted regulator of complement/ECM) was also not significantly affected by MW151 treatment but showed a main effect of sex (*F*[1, 66] = 8.91, *p =* 0.004, partial η^2^ = 0.119; Figure 8E). While there was a visual pattern for an interaction of treatment by sex, with female MW151‐treated mice appearing to have more *LCN2* and *PTX3* than the other groups, the interaction was not statistically significant (Table S8).

Microglia and macrophage markers were also unaffected by MW151. Microglia/macrophage endpoints were selected to index recruitment/activation and disease‐associated microglia features, including CD45 (pan‐leukocyte marker reporting parenchymal macrophages/activated microglia), CLEC7A/Dectin‐1 (DAM‐associated phagocytic receptor), CD68 (lysosomal/phagolysosomal activity), and *TREM2* (microglial receptor linked to plaque‐associated/DAM programs). In the neocortex, the percent area stained for CD45 (Figure 8F–G) and Dectin‐1 (Figure 8H,I) did not differ significantly by MW151 treatment. Cluster density analysis across size bins for both Dectin‐1 and CD45 also showed no significant treatment effects (Table S9), confirming that MW151 did not alter either total immunoreactivity or cellular distribution patterns. *CD68* and *TREM2* transcripts also showed no significant treatment effect but exhibited robust sex main effects for CD68 (*F*[1, 66] = 11.62, *p =* 0.001, partial η^2^ = 0.150; Figure 8J) and *TREM2* (*F*[1, 66] = 9.03, *p =* 0.004, partial η^2^ = 0.120; Figure 8K).

These findings extend MW151's cytokine‐suppressing profile to the SAA model. The absence of a detectable IL‐1β reduction at this dose/time could reflect higher baseline IL‐1β in this model or lower in vivo sensitivity of IL‐1β to MW151 under these conditions; both are compatible with the observed sleep effects and the selective TNF‐α reduction. hAPP^WT^ KI mice were included in the intervention cohort at lower numbers, as these animals lack the sleep and inflammatory phenotypes targeted by MW151. Consistent with this, MW151 had minimal effects in WT mice (Tables S10–S14).

## 4 DISCUSSION

Sleep and circadian disturbances are among the earliest symptoms of AD. In our longitudinal profiling of hAPP^SAA^ KI mice, changes in light–phase sleep and circadian stability tracked with rising Aβ burden and an increasingly proinflammatory milieu. MW151 treatment, a brain‐penetrant modulator of glia‐mediated cytokine overproduction, in 15‐month‐old SAA mice increased light‐phase sleep, decreased fragmentation (MESOR), and lowered cortical TNF‐α without altering Aβ40/42 or total daily sleep. These findings identify excessive glial cytokine signaling as a contributor to AD‐related sleep–wake instability and elevate sleep restoration as a translatable biomarker of target engagement.

Our behavioral characterization is consistent with and expands upon recent work on the hAPP^SAA^ KI model. Lu et al.^28^ reported contextual and cued fear‐conditioning deficits in 12 to 13.5 months old hAPP^SAA^ KI mice when compared to WT B6 controls; however, when compared directly to their congenic hAPP^WT^ KI line, no significant impairments were observed. Both studies point to more subtle or circuit‐specific vulnerabilities, such as female‐biased circadian disruption or impaired cognitive flexibility, that emerge in midlife. Similarly, Blackmer‐Raynolds et al.^29^ observed normal Y‐maze, Barnes maze, and object‐location performance at 12 months, with measurable impairments only emerging at 16 months. Nesting impairments were evident in both sexes, reflecting progressive deterioration of daily‐living behaviors, while reversal‐learning impairments were modest and selective, consistent with clinical evidence that executive dysfunction and circadian instability often precede overt memory decline.^10^, ^58^, ^59^ Our circadian results complement longitudinal home‐cage activity data from Xia et al.,^27^ who demonstrated that female SAA mice developed dark‐phase hyperactivity and increased wheel‐running beginning around 8 months before overt amyloid deposition. This increase in nocturnal activity corresponds to the greater rhythm fragmentation and reduced stability we observed in females, suggesting a breakdown in circadian control mechanisms that begins early and progresses with pathology. The use of non‐invasive piezoelectric sleep monitoring enabled the assessment of sleep–wake timing and consolidation versus fragmentation of sleep throughout the lifespan in this study. Piezoelectric cages have been shown to provide sleep measures comparable to and validated against electroencephalogram (EEG)/electromyography (EMG) measures^34^, ^35^, ^36^; however, future EEG/EMG studies would be ideal to assess specific sleep parameters in greater detail, including rapid eye movement (REM), non‐REM (NREM), EEG delta power, and more and could both confirm and extend these findings.

Both sexes accumulated Aβ with age, yet females showed greater sleep and circadian disruption beginning at midlife, consistent with other amyloidosis models.^60^ Notably, despite robust sex differences in sleep fragmentation, amyloid burden and glial activation did not differ between sexes, suggesting female vulnerability reflects circuit‐level sensitivity to neuroinflammatory signaling rather than greater pathological burden. The absence of sex differences in pathology may reflect an advantage of KI models: Many reported sex effects in transgenic APP mice are driven by estrogen‐responsive elements in transgene promoters (e.g., Thy1, PrP).^44^ The hAPP^SAA^ KI mice express APP under the endogenous promoter, eliminating this confound. The hAPP^SAA^ model expresses APP under the endogenous promoter, eliminating this confound and providing physiological relevance for understanding sleep–wake transitions in AD. This pattern mirrors human observations, with females showing stronger correlations between wake after sleep onset and cortical thinning in AD‐vulnerable regions^61^ and more pronounced circadian disruption despite similar biomarker profiles.^19^, ^61^, ^62^ Circadian fragmentation predicts faster cognitive decline and AD progression,^63^ with associations generally stronger in women.^64^, ^65^ The underlying mechanisms may involve loss of subcortical wake‐promoting neurons,^66^ and while some biomarker relationships show male‐specific vulnerabilities,^67^ the overall pattern suggests females are more susceptible to the cognitive consequences of sleep and circadian disruption in AD.

The temporal alignment of sleep disruption onset in female mice with reproductive senescence (9 to 12 months) suggests hormonal modulation may contribute to the observed sex differences, though we did not directly measure estrous cycling or hormone levels in this study. If reproductive transitions play a role, the mechanism could involve estradiol's established effects on both circadian and inflammatory systems. Declining estradiol coincides with increased inflammatory tone, altered circadian function, and Aβ deposition.^68^, ^69^ Estradiol supports circadian amplitude^70^, ^71^ and promotes anti‐inflammatory glial states^72^, ^73^, ^74^ through classical estrogen receptors and GPER,^75^ raising the possibility that loss of these regulatory effects amplifies cytokine responses to Aβ during a vulnerable window.^76^ However, sex differences in baseline glial phenotypes or sleep‐promoting circuits may contribute independently of hormones, and our data cannot distinguish between these alternatives.

Beyond these sex differences, the temporal dynamics of sleep disruption reveal changes in disease progression. We found that sleep and circadian disturbances emerged months before cognitive deficits appeared in the hAPP^SAA^ KI model. This sequence aligns with human cohort data showing that sleep fragmentation in cognitively normal older adults predicts incident AD over subsequent years,^2^ with long sleep latency (>30 min) predicting cognitive decline^77^ and excessive daytime sleepiness accelerating Aβ accumulation in AD‐vulnerable brain regions.^78^ A recent meta‐analysis confirmed these associations, showing that sleep disorders, including insomnia (hazard ratio [HR] 1.36) and obstructive sleep apnea (HR 1.45), significantly increase AD risk across multiple cohort studies.^7^ The temporal precedence positions sleep disruption as a potential early biomarker and intervention target rather than a late consequence of neurodegeneration.

Given that neuroinflammation is a hallmark of AD pathology, cytokines may provide a mechanistic link between disease progression and sleep disruption. Indeed, sleep deprivation elevates IL‐1β, TNF‐α, and IL‐6 in WT rodents, exogenous cytokines disrupt sleep–wake patterns, and blocking cytokine signaling improves sleep in inflammatory conditions.^79^ Our contribution extends this cytokine–sleep relationship to a physiologically relevant AD model, demonstrating that cytokine‐mediated sleep disruption occurs during early pathology and is reversible through pharmacologic intervention. Cytokines such as IL‐1β and TNF‐α are known to acutely promote sleep, acting as endogenous sleep‐regulatory molecules under physiological conditions.^30^, ^31^, ^32^, ^52^, ^56^ Importantly, the effects of cytokines on sleep are dose‐dependent. For example, low doses of IL‐1 increase NREM sleep, while higher doses inhibit it.^79^ This suggests that the progressive elevation of cytokines during AD pathogenesis may drive the transition from compensatory sleep changes to pathological sleep disruption. This dose dependency suggests that therapeutic approaches should aim to normalize, rather than eliminate, cytokine signaling to restore healthy sleep patterns in AD.

Using MW151 (now in Phase I human testing, NCT04120233), a brain‐penetrant small molecule that selectively suppresses glia‐mediated cytokine overproduction without depressing physiological cytokine signaling, we demonstrate that normalizing cytokine tone partially restores sleep–wake patterns even in aged animals.^33^, ^39^, ^40^, ^45^, ^46^, ^47^, ^48^, ^49^, ^50^, ^51^ Although the sleep phenotype in hAPP^SAA^ KI mice was more pronounced in females, MW151 increased light‐phase sleep comparably in both sexes, with no treatment × sex interaction. These findings indicate that treatment effects did not differ statistically by sex within the SAA cohort. Our results parallel and extend prior MW151 work in the APP/PS1 model, where the same low dose (2.5 mg/kg) reduced cortical IL‐1β, attenuated microgliosis, rescued hippocampal Long‐term potentiation, and preserved synaptic proteins without altering Aβ burden.^33^ Similarly, APP^swe^/PS1^dE9^ mice exhibit NREM deficits and sleep fragmentation by 6 months, and optogenetic enhancement of slow‐wave activity or astrocyte function restored sleep architecture and reduced amyloid burden.^80^, ^81^ The present findings broaden the functional scope of MW151 by showing that glial cytokine normalization also improves circadian and sleep integrity. Both studies converge on a shared mechanism, where MW151 resets glial cytokine output, dampening pathological but not homeostatic inflammatory signaling, thereby improving network performance – whether synaptic or circadian. The fact that MW151 was effective even when administered at 15 months, well after pathology onset, further supports the reversibility of cytokine‐driven functional impairments and underscores the potential of cytokine modulation as a therapeutic approach even in later disease stages. The rapid normalization of sleep within 2 weeks of MW151 treatment suggests that elevated cytokine tone exerts acute and reversible effects on sleep circuits. This aligns with evidence that sleep deprivation increases interstitial Aβ,^82^ while sleep promotes glymphatic clearance,^83^ supporting a feedforward cycle where Aβ drives cytokine elevation, cytokines disrupt sleep, and disrupted sleep impairs Aβ clearance. However, our cytokine profiling focused on the cortex, where amyloid plaques are most abundant and where prior studies have shown inflammation. Whether cortical inflammation drives changes in sleep circuits through descending projections or circulating factors, or instead reflects parallel processes in sleep‐regulatory regions, remains to be established. Profiling the sleep‐promoting ventrolateral preoptic area, the circadian pacemaker (suprachiasmatic nucleus), and arousal centers alongside the cortex will clarify regional specificity. Only one MW151 dose and start age were tested in this study. Future studies should explore different dosing and treatment regimens to determine the optimal treatment paradigm, which could help inform the translation of the findings to people.

In summary, this work identifies glial inflammation as a modifiable contributor to AD‐related sleep and circadian disruption. Moreover, sleep parameters may serve as pharmacodynamic endpoints to assess target engagement of anti‐inflammatory interventions, though this relationship requires further validation. The rapid improvement with cytokine normalization, coupled with preserved amyloid burden, points to a therapeutic lever acting on network function without requiring changes in plaque load. The sex‐ and timing‐dependent pattern highlights a biologically defined window for intervention, outlining a path from mechanism to trial design using objective sleep metrics.

## AUTHOR CONTRIBUTIONS

A.D.B., T.M., and M.J.D. designed the study. M.P.M., M.J.D., B.F.O., S.S., and A.D.B. provided funding and input on design. L.J.V.E. provided reagents and input on design. T.M. led experimental work with contributions from M.R.H., C.E.J., M.G.L., H.R.W., S.S., M.F.C., K.N.R., L.B., H.M.H., O.A.A.E., M.A.E., M.J.D., and K.K. A.D.B. conducted statistical analyses. T.M. and A.D.B. drafted the manuscript. All authors contributed to data interpretation, critically revised the manuscript, approved the final version, and accept responsibility for its accuracy and integrity.

## CONFLICT OF INTEREST STATEMENT

LJVE is an inventor on patents covering MW151 and a scientific founder of ImmunoChem Therapeutics, LLC, a start‐up formed to commercialize MW151. BFO is a co‐founder and co‐owner of Signal Solutions LLC, which makes the PiezoSleep technology used here. The other authors declare no competing financial interests or conflicts of interest related to this manuscript. Author disclosures are available in the Supporting Information.

## FUNDING INFORMATION

Funding for this study was provided by the National Institutes of Health (R01 AG068215; MPM, MJD, ADB, BFO, SS).

## CONSENT STATEMENT

This study did not involve human subjects; therefore, informed consent was not required.

## DECLARATION OF GENERATIVE AI USAGE

ChatGPT and Claude were used to assist with language editing. The authors reviewed and approved all content, and they take full responsibility for the final manuscript.

## Supporting information



Supporting Information

Supporting Information

Supporting Information

## References

[1] Guarnieri B , Adorni F , Musicco M , et al. Prevalence of sleep disturbances in mild cognitive impairment and dementing disorders: a multicenter Italian clinical cross‐sectional study on 431 patients. Dement Geriatr Cogn Disord. 2012;33(1):50‐58. doi:10.1159/000335363 22415141 10.1159/000335363 PMC3696366

[2] Lim AS , Kowgier M , Yu L , Buchman AS , Bennett DA . Sleep Fragmentation and the Risk of Incident Alzheimer's Disease and Cognitive Decline in Older Persons. Sleep. 2013;36(7):1027‐1032. doi:10.5665/sleep.2802 23814339 10.5665/sleep.2802 PMC3669060

[3] Hahn EA , Wang HX , Andel R , Fratiglioni L . A change in sleep pattern may predict Alzheimer disease. Am J Geriatr Psychiatry. 2014;22(11):1262‐1271. doi:10.1016/j.jagp.2013.04.015 23954041 10.1016/j.jagp.2013.04.015

[4] Han Z , Yang X , Huang S . Sleep deprivation: a risk factor for the pathogenesis and progression of Alzheimer's disease. Heliyon. 2024;10(7):e28819. doi:10.1016/j.heliyon.2024.e28819 38623196 10.1016/j.heliyon.2024.e28819 PMC11016624

[5] Wang S , Zheng X , Huang J , Liu J , Li C , Shang H . Sleep characteristics and risk of Alzheimer's disease: a systematic review and meta‐analysis of longitudinal studies. J Neurol. 2024;271(7):3782‐3793. doi:10.1007/s00415‐024‐12380‐7 38656621 10.1007/s00415-024-12380-7

[6] McConnell BV , Deng Y , Lucey BP . Sleep and Neurodegeneration: examining Potential Physiological Mechanisms. Curr Sleep Med Rep. 2025;11(1):10. doi:10.1007/s40675‐024‐00316‐6 10.1007/s40675-024-00316-6 PMC12372957 40862083

[7] Ungvari Z , Fekete M , Lehoczki A , et al. Sleep disorders increase the risk of dementia, Alzheimer's disease, and cognitive decline: a meta‐analysis. Geroscience. 2025;47(3):4899‐4920. doi:10.1007/s11357‐025‐01637‐2 40214959 10.1007/s11357-025-01637-2 PMC12181552

[8] Moran M , Lynch CA , Walsh C , Coen R , Coakley D , Lawlor BA . Sleep disturbance in mild to moderate Alzheimer's disease. Sleep Med. 2005;6(4):347‐352. doi:10.1016/j.sleep.2004.12.005 15978517 10.1016/j.sleep.2004.12.005

[9] Rothman SM , Mattson MP . Sleep disturbances in Alzheimer's and Parkinson's diseases. Neuromolecular Med. 2012;14(3):194‐204. doi:10.1007/s12017‐012‐8181‐2 22552887 10.1007/s12017-012-8181-2 PMC4544709

[10] Musiek ES , Bhimasani M , Zangrilli MA , Morris JC , Holtzman DM , Ju YS . Circadian Rest‐Activity Pattern Changes in Aging and Preclinical Alzheimer Disease. JAMA Neurol. 2018;75(5):582‐590. doi:10.1001/jamaneurol.2017.4719 29379963 10.1001/jamaneurol.2017.4719 PMC5885197

[11] Winer JR , Lok R , Weed L , et al. Impaired 24‐h activity patterns are associated with an increased risk of Alzheimer's disease, Parkinson's disease, and cognitive decline. Alzheimers Res Ther. 2024;16(1):35. doi:10.1186/s13195‐024‐01411‐0 38355598 10.1186/s13195-024-01411-0 PMC10865579

[12] Gramkow MH , Brink‐Kjaer A , Clemmensen FK , et al. Diagnostic performance of actigraphy in Alzheimer's disease using a machine learning classifier—a cross‐sectional memory clinic study. Alzheimers Res Ther. 2025;17(1):111. doi:10.1186/s13195‐025‐01751‐5 40399918 10.1186/s13195-025-01751-5 PMC12374425

[13] Iliff JJ , Wang M , Liao Y , et al. A paravascular pathway facilitates CSF flow through the brain parenchyma and the clearance of interstitial solutes, including amyloid beta. Sci Transl Med. 2012;4(147):147ra111. doi:10.1126/scitranslmed.3003748 10.1126/scitranslmed.3003748 PMC3551275 22896675

[14] Wang C , Holtzman DM . Bidirectional relationship between sleep and Alzheimer's disease: role of amyloid, tau, and other factors. Neuropsychopharmacology. 2020;45(1):104‐120. doi:10.1038/s41386‐019‐0478‐5 31408876 10.1038/s41386-019-0478-5 PMC6879647

[15] Duncan MJ , Guerriero LE , Kohler K , et al. Chronic Fragmentation of the Daily Sleep‐Wake Rhythm Increases Amyloid‐beta Levels and Neuroinflammation in the 3xTg‐AD Mouse Model of Alzheimer's Disease. Neuroscience. 2022;481:111‐122. doi:10.1016/j.neuroscience.2021.11.042 34856352 10.1016/j.neuroscience.2021.11.042 PMC8941625

[16] Liu S , Liu X , Ke M , Wang J . Sleep fragmentation impairs cognitive function and exacerbates Alzheimer's disease‐related pathology in a mouse model by disrupting mitochondrial biogenesis. Exp Neurol. 2025;386:115153. doi:10.1016/j.expneurol.2025.115153 39832661 10.1016/j.expneurol.2025.115153

[17] Keil SA , Jansson D , Braun M , Iliff JJ . Glymphatic dysfunction in Alzheimer's disease: a critical appraisal. Science. 2025;389(6756):eadv8269. doi:10.1126/science.adv8269 40638744 10.1126/science.adv8269

[18] Turton SM , Padgett S , Maisel MT , et al. Interactions between daily sleep‐wake rhythms, gamma‐secretase, and amyloid‐beta peptide pathology point to complex underlying relationships. Biochim Biophys Acta Mol Basis Dis. 2025;1871(6):167840. doi:10.1016/j.bbadis.2025.167840 40222459 10.1016/j.bbadis.2025.167840 PMC12186295

[19] Ferretti MT , Iulita MF , Cavedo E , et al. Sex differences in Alzheimer disease—the gateway to precision medicine. Nat Rev Neurol. 2018;14(8):457‐469. doi:10.1038/s41582‐018‐0032‐9 29985474 10.1038/s41582-018-0032-9

[20] Maki PM , Jaff NG . Menopause and brain fog: how to counsel and treat midlife women. Menopause. 2024;31(7):647‐649. doi:10.1097/GME.0000000000002382 38888619 10.1097/GME.0000000000002382

[21] Mishra A , Shang Y , Wang Y , Bacon ER , Yin F , Brinton RD . Dynamic Neuroimmune Profile during Mid‐life Aging in the Female Brain and Implications for Alzheimer Risk. iScience. 2020;23(12):101829. doi:10.1016/j.isci.2020.101829 33319170 10.1016/j.isci.2020.101829 PMC7724165

[22] Dohm‐Hansen S , English JA , Lavelle A , Fitzsimons CP , Lucassen PJ , Nolan YM . The ‘middle‐aging’ brain. Trends Neurosci. 2024;47(4):259‐272. doi:10.1016/j.tins.2024.02.001 38508906 10.1016/j.tins.2024.02.001

[23] Martin SC , Joyce KK , Lord JS , et al. Sleep Disruption Precedes Forebrain Synaptic Tau Burden and Contributes to Cognitive Decline in a Sex‐Dependent Manner in the P301S Tau Transgenic Mouse Model. eNeuro. 2024;11(6):ENEURO.0004‐0024.2024. doi:10.1523/ENEURO.0004‐24.2024 38858068 10.1523/ENEURO.0004-24.2024 PMC11209651

[24] Sethi M , Joshi SS , Webb RL , et al. Increased fragmentation of sleep‐wake cycles in the 5XFAD mouse model of Alzheimer's disease. Neuroscience. 2015;290:80‐89. doi:10.1016/j.neuroscience.2015.01.035 25637807 10.1016/j.neuroscience.2015.01.035 PMC4361816

[25] Drew VJ , Wang C , Kim T . Progressive sleep disturbance in various transgenic mouse models of Alzheimer's disease. Front Aging Neurosci. 2023;15:1119810. doi:10.3389/fnagi.2023.1119810 37273656 10.3389/fnagi.2023.1119810 PMC10235623

[26] Jankowsky JL , Zheng H . Practical considerations for choosing a mouse model of Alzheimer's disease. Mol Neurodegener. 2017;12(1):89. doi:10.1186/s13024‐017‐0231‐7 29273078 10.1186/s13024-017-0231-7 PMC5741956

[27] Xia D , Lianoglou S , Sandmann T , et al. Novel App knock‐in mouse model shows key features of amyloid pathology and reveals profound metabolic dysregulation of microglia. Mol Neurodegener. 2022;17(1):41. doi:10.1186/s13024‐022‐00547‐7 35690868 10.1186/s13024-022-00547-7 PMC9188195

[28] Lu W , Shue F , Kurti A , et al. Amyloid pathology and cognitive impairment in hAbeta‐KI and APP(SAA)‐KI mouse models of Alzheimer's disease. Neurobiol Aging. 2025;145:13‐23. doi:10.1016/j.neurobiolaging.2024.10.005 39447490 10.1016/j.neurobiolaging.2024.10.005 PMC11578766

[29] Blackmer‐Raynolds L , Lipson LD , Fraccaroli I , Krout IN , Chang J , Sampson TR . Longitudinal characterization reveals behavioral impairments in aged APP knock in mouse models. Sci Rep. 2025;15(1):4631. doi:10.1038/s41598‐025‐89051‐8 39920176 10.1038/s41598-025-89051-8 PMC11805898

[30] Opp MR , Krueger JM . Sleep and immunity: a growing field with clinical impact. Brain Behav Immun. 2015;47:1‐3. doi:10.1016/j.bbi.2015.03.011 25849976 10.1016/j.bbi.2015.03.011 PMC4685944

[31] Krueger JM , Majde JA , Rector DM . Cytokines in immune function and sleep regulation. Handb Clin Neurol. 2011;98:229‐240. doi:10.1016/B978‐0‐444‐52006‐7.00015‐0 21056190 10.1016/B978-0-444-52006-7.00015-0 PMC5440845

[32] KRUEGER JM , FANG J , TAISHI P , CHEN Z , KUSHIKATA T , GARDI J . Sleep: a Physiologic Role for IL‐1β and TNF‐α. Annals of the New York Academy of Sciences. 1998;856(1):148‐159. doi:10.1111/j.1749‐6632.1998.tb08323.x 9917875 10.1111/j.1749-6632.1998.tb08323.x

[33] Bachstetter AD , Norris CM , Sompol P , et al. Early stage drug treatment that normalizes proinflammatory cytokine production attenuates synaptic dysfunction in a mouse model that exhibits age‐dependent progression of Alzheimer's disease‐related pathology. J Neurosci. 2012;32(30):10201‐10210. doi:10.1523/JNEUROSCI.1496‐12.2012 22836255 10.1523/JNEUROSCI.1496-12.2012 PMC3419360

[34] Yaghouby F , Donohue KD , O'Hara BF , Sunderam S . Noninvasive dissection of mouse sleep using a piezoelectric motion sensor. J Neurosci Methods. 2016;259:90‐100. doi:10.1016/j.jneumeth.2015.11.004 26582569 10.1016/j.jneumeth.2015.11.004 PMC4715949

[35] Mang GM , Nicod J , Emmenegger Y , Donohue KD , O'Hara BF , Franken P . Evaluation of a piezoelectric system as an alternative to electroencephalogram/electromyogram recordings in mouse sleep studies. Sleep. 2014;37(8):1383‐1392. doi:10.5665/sleep.3936 25083019 10.5665/sleep.3936 PMC4096208

[36] Donohue KD , Medonza DC , Crane ER , O'Hara BF . Assessment of a non‐invasive high‐throughput classifier for behaviours associated with sleep and wake in mice. Biomed Eng Online. 2008;7:14. doi:10.1186/1475‐925X‐7‐14 18405376 10.1186/1475-925X-7-14 PMC2365952

[37] Deacon RM . Assessing nest building in mice. Nat Protoc. 2006;1(3):1117‐1119. doi:10.1038/nprot.2006.170 17406392 10.1038/nprot.2006.170

[38] Macheda T , Roberts KN , Morganti JM , Braun DJ , Bachstetter AD . Optimization and validation of a modified radial‐arm water maze protocol using a murine model of mild closed head traumatic brain injury. PLoS One. 2020;15(8):e0232862. doi:10.1371/journal.pone.0232862 32810143 10.1371/journal.pone.0232862 PMC7433858

[39] Bachstetter AD , Webster SJ , Goulding DS , Morton JE , Watterson DM , Van Eldik LJ . Attenuation of traumatic brain injury‐induced cognitive impairment in mice by targeting increased cytokine levels with a small molecule experimental therapeutic. J Neuroinflammation. 2015;12:69. doi:10.1186/s12974‐015‐0289‐5 25886256 10.1186/s12974-015-0289-5 PMC4396836

[40] Webster SJ , Van Eldik LJ , Watterson DM , Bachstetter AD . Closed head injury in an age‐related Alzheimer mouse model leads to an altered neuroinflammatory response and persistent cognitive impairment. J Neurosci. 2015;35(16):6554‐6569. doi:10.1523/JNEUROSCI.0291‐15.2015 25904805 10.1523/JNEUROSCI.0291-15.2015 PMC4405562

[41] Hawkins MR WH , Johnson CE , Macheda T et al.. Chronic Sleep Fragmentation Differentially Affects Alzheimer's Disease Pathology in Male and Female APPSAA Knock‐in Mice. Journal of Inflammation Research. 2025;18:14325‐14341. 41126970 10.2147/JIR.S544625 PMC12537530

[42] Macheda T , Roberts K , Lyons DN , et al. Chronic Intermittent Hypoxia Induces Robust Astrogliosis in an Alzheimer's Disease‐Relevant Mouse Model. Neuroscience. 2019;398:55‐63. doi:10.1016/j.neuroscience.2018.11.040 30529693 10.1016/j.neuroscience.2018.11.040 PMC6402802

[43] Cohen J . Statistical Power Analysis for the Behavioral Sciences. Lawrence Erlbaum Associates. 1988.

[44] Neuharth JI , Hernandez KS , Bernholtz J , Edwards H , Stewart A . Consideration of sex as a biological variable over the history of the 5xFAD Alzheimer's Disease mouse model. Biol Sex Differ. 2025;16(1):105. doi:10.1186/s13293‐025‐00788‐3 41408378 10.1186/s13293-025-00788-3 PMC12709748

[45] Braun DJ , Powell DK , McLouth CJ , Roy SM , Watterson DM , Van Eldik LJ . Therapeutic treatment with the anti‐inflammatory drug candidate MW151 may partially reduce memory impairment and normalizes hippocampal metabolic markers in a mouse model of comorbid amyloid and vascular pathology. PLoS One. 2022;17(1):e0262474. doi:10.1371/journal.pone.0262474 35081152 10.1371/journal.pone.0262474 PMC8791470

[46] Zhou Z , Bachstetter AD , Spani CB , Roy SM , Watterson DM , Van Eldik LJ . Retention of normal glia function by an isoform‐selective protein kinase inhibitor drug candidate that modulates cytokine production and cognitive outcomes. J Neuroinflammation. 2017;14(1):75. doi:10.1186/s12974‐017‐0845‐2 28381303 10.1186/s12974-017-0845-2 PMC5382362

[47] Bachstetter AD , Zhou Z , Rowe RK , et al. MW151 Inhibited IL‐1beta Levels after Traumatic Brain Injury with No Effect on Microglia Physiological Responses. PLoS One. 2016;11(2):e0149451. doi:10.1371/journal.pone.0149451 26871438 10.1371/journal.pone.0149451 PMC4752278

[48] Somera‐Molina KC , Nair S , Van Eldik LJ , Watterson DM , Wainwright MS . Enhanced microglial activation and proinflammatory cytokine upregulation are linked to increased susceptibility to seizures and neurologic injury in a ‘two‐hit’ seizure model. Brain Res. 2009;1282:162‐172. doi:10.1016/j.brainres.2009.05.073 19501063 10.1016/j.brainres.2009.05.073 PMC2739829

[49] Karpus WJ , Reynolds N , Behanna HA , Van Eldik LJ , Watterson DM . Inhibition of experimental autoimmune encephalomyelitis by a novel small molecular weight proinflammatory cytokine suppressing drug. J Neuroimmunol. 2008;203(1):73‐78. doi:10.1016/j.jneuroim.2008.06.039 18678415 10.1016/j.jneuroim.2008.06.039 PMC2614915

[50] Somera‐Molina KC , Robin B , Somera CA , et al. Glial activation links early‐life seizures and long‐term neurologic dysfunction: evidence using a small molecule inhibitor of proinflammatory cytokine upregulation. Epilepsia. 2007;48(9):1785‐1800. doi:10.1111/j.1528‐1167.2007.01135.x 17521344 10.1111/j.1528-1167.2007.01135.x

[51] Hu W , Ralay Ranaivo H , Roy SM , et al. Development of a novel therapeutic suppressor of brain proinflammatory cytokine up‐regulation that attenuates synaptic dysfunction and behavioral deficits. Bioorg Med Chem Lett. 2007;17(2):414‐418. doi:10.1016/j.bmcl.2006.10.028 17079143 10.1016/j.bmcl.2006.10.028 PMC1868432

[52] Krueger JM , Obal FJ , Fang J , Kubota T , Taishi P . The role of cytokines in physiological sleep regulation. Ann N Y Acad Sci. 2001;933:211‐221. doi:10.1111/j.1749‐6632.2001.tb05826.x 12000022 10.1111/j.1749-6632.2001.tb05826.x

[53] Rockstrom MD , Chen L , Taishi P , et al. Tumor necrosis factor alpha in sleep regulation. Sleep Med Rev. 2018;40:69‐78. doi:10.1016/j.smrv.2017.10.005 29153862 10.1016/j.smrv.2017.10.005 PMC5955790

[54] Zielinski MR , Gibbons AJ . Neuroinflammation, Sleep, and Circadian Rhythms. Front Cell Infect Microbiol. 2022;12:853096. doi:10.3389/fcimb.2022.853096 35392608 10.3389/fcimb.2022.853096 PMC8981587

[55] Prolo P , Iribarren FJ , Negaos N , Chiappelli F . Role of Pro‐Inflammatory Cytokines in Sleep Disorders. In: Cardinali DP , Pandi‐Perumal SR , eds. Neuroendocrine Correlates of Sleep/Wakefulness. Springer US; 2005:537‐552.

[56] Krueger JM , Clinton JM , Winters BD , et al. Involvement of cytokines in slow wave sleep. Prog Brain Res. 2011;193:39‐47. doi:10.1016/B978‐0‐444‐53839‐0.00003‐X 21854954 10.1016/B978-0-444-53839-0.00003-X PMC3645329

[57] Veler H . Sleep and Inflammation: bidirectional Relationship. Sleep Med Clin. 2023;18(2):213‐218. doi:10.1016/j.jsmc.2023.02.003 37120163 10.1016/j.jsmc.2023.02.003

[58] Harrington MG , Chiang J , Pogoda JM , et al. Executive function changes before memory in preclinical Alzheimer's pathology: a prospective, cross‐sectional, case control study. PLoS One. 2013;8(11):e79378. doi:10.1371/journal.pone.0079378 24260210 10.1371/journal.pone.0079378 PMC3832547

[59] Grober E , Hall CB , Lipton RB , Zonderman AB , Resnick SM , Kawas C . Memory impairment, executive dysfunction, and intellectual decline in preclinical Alzheimer's disease. J Int Neuropsychol Soc. 2008;14(2):266‐278. doi:10.1017/S1355617708080302 18282324 10.1017/S1355617708080302 PMC2763488

[60] Kim KJ , Villegas AL , Kelley AR , et al. Sex differences in sleep fragmentation in 5xFAD mice. Neuroscience. 2025;589:118‐127. doi:10.1016/j.neuroscience.2025.10.035 41138967 10.1016/j.neuroscience.2025.10.035

[61] Stankeviciute L , Tort‐Colet N , Fernandez‐Arcos A , et al. Associations between objective sleep metrics and brain structure in cognitively unimpaired adults: interactions with sex and Alzheimer's biomarkers. Alzheimers Dement. 2025;21(6):e70353. doi:10.1002/alz.70353 40566790 10.1002/alz.70353 PMC12198473

[62] Price BR , Walker KA , Eissman JM , et al. Sex differences and the role of estrogens in the immunological underpinnings of Alzheimer's disease. Alzheimers Dement (N Y). 2025;11(3):e70139. doi:10.1002/trc2.70139 40827126 10.1002/trc2.70139 PMC12358009

[63] Li P , Gao L , Gaba A , et al. Circadian disturbances in Alzheimer's disease progression: a prospective observational cohort study of community‐based older adults. Lancet Healthy Longev. 2020;1(3):e96‐e105. doi:10.1016/s2666‐7568(20)30015‐5 34179863 10.1016/s2666-7568(20)30015-5 PMC8232345

[64] Rabinowitz JA , An Y , He L , et al. Associations of circadian rest/activity rhythms with cognition in middle‐aged and older adults: demographic and genetic interactions. Front Neurosci. 2022;16:952204. doi:10.3389/fnins.2022.952204 36312032 10.3389/fnins.2022.952204 PMC9597505

[65] Chen JC , Espeland MA , Brunner RL , et al. Sleep duration, cognitive decline, and dementia risk in older women. Alzheimers Dement. 2016;12(1):21‐33. doi:10.1016/j.jalz.2015.03.004 26086180 10.1016/j.jalz.2015.03.004 PMC4679723

[66] Oh JY , Walsh CM , Ranasinghe K , et al. Subcortical Neuronal Correlates of Sleep in Neurodegenerative Diseases. JAMA Neurol. 2022;79(5):498‐508. doi:10.1001/jamaneurol.2022.0429 35377391 10.1001/jamaneurol.2022.0429 PMC8981071

[67] Van Egroo M , Beckers E , Ashton NJ , Blennow K , Zetterberg H , Jacobs HIL . Sex differences in the relationships between 24‐h rest‐activity patterns and plasma markers of Alzheimer's disease pathology. Alzheimers Res Ther. 2024;16(1):277. doi:10.1186/s13195‐024‐01653‐y 39736697 10.1186/s13195-024-01653-y PMC11684129

[68] Ikuta K , Ejima A , Abe S , Shimba A . Control of immunity and allergy by steroid hormones. Allergol Int. 2022;71(4):432‐436. doi:10.1016/j.alit.2022.07.006 35973911 10.1016/j.alit.2022.07.006

[69] Mosconi L , Berti V , Dyke J , et al. Menopause impacts human brain structure, connectivity, energy metabolism, and amyloid‐beta deposition. Sci Rep. 2021;11(1):10867. doi:10.1038/s41598‐021‐90084‐y 34108509 10.1038/s41598-021-90084-y PMC8190071

[70] Schwartz MD , Mong JA . Estradiol modulates recovery of REM sleep in a time‐of‐day‐dependent manner. Am J Physiol Regul Integr Comp Physiol. 2013;305(3):R271‐80. doi:10.1152/ajpregu.00474.2012 23678032 10.1152/ajpregu.00474.2012 PMC3743004

[71] Rahman SA , Grant LK , Gooley JJ , Rajaratnam SMW , Czeisler CA , Lockley SW . Endogenous Circadian Regulation of Female Reproductive Hormones. J Clin Endocrinol Metab. 2019;104(12):6049‐6059. doi:10.1210/jc.2019‐00803 31415086 10.1210/jc.2019-00803 PMC6821202

[72] Villa A , Vegeto E , Poletti A , Maggi A . Estrogens, Neuroinflammation, and Neurodegeneration. Endocr Rev. 2016;37(4):372‐402. doi:10.1210/er.2016‐1007 27196727 10.1210/er.2016-1007 PMC4971309

[73] Butler MJ , Perrini AA , Eckel LA . Estradiol treatment attenuates high fat diet‐induced microgliosis in ovariectomized rats. Horm Behav. 2020;120:104675. doi:10.1016/j.yhbeh.2020.104675 31923417 10.1016/j.yhbeh.2020.104675 PMC7117977

[74] Loiola RA , Wickstead ES , Solito E , McArthur S . Estrogen Promotes Pro‐resolving Microglial Behavior and Phagocytic Cell Clearance Through the Actions of Annexin A1. Front Endocrinol (Lausanne). 2019;10:420. doi:10.3389/fendo.2019.00420 31297095 10.3389/fendo.2019.00420 PMC6607409

[75] Zhao TZ , Ding Q , Hu J , He SM , Shi F , Ma LT . GPER expressed on microglia mediates the anti‐inflammatory effect of estradiol in ischemic stroke. Brain Behav. 2016;6(4):e00449. doi:10.1002/brb3.449 27127723 10.1002/brb3.449 PMC4840664

[76] Martinez‐Muniz GA , Wood SK . Sex Differences in the Inflammatory Consequences of Stress: implications for Pharmacotherapy. J Pharmacol Exp Ther. 2020;375(1):161‐174. doi:10.1124/jpet.120.266205 32759370 10.1124/jpet.120.266205 PMC7569308

[77] Suh SW , Han JW , Lee JR , et al. Sleep and cognitive decline: a prospective nondemented elderly cohort study. Ann Neurol. 2018;83(3):472‐482. doi:10.1002/ana.25166 29394505 10.1002/ana.25166

[78] Carvalho DZ , St Louis EK , Knopman DS , et al. Association of Excessive Daytime Sleepiness With Longitudinal beta‐Amyloid Accumulation in Elderly Persons Without Dementia. JAMA Neurol. 2018;75(6):672‐680. doi:10.1001/jamaneurol.2018.0049 29532057 10.1001/jamaneurol.2018.0049 PMC5885188

[79] Irwin MR . Sleep and inflammation: partners in sickness and in health. Nat Rev Immunol. 2019;19(11):702‐715. doi:10.1038/s41577‐019‐0190‐z 31289370 10.1038/s41577-019-0190-z

[80] Zhao Q , Maci M , Miller MR , et al. Sleep restoration by optogenetic targeting of GABAergic neurons reprograms microglia and ameliorates pathological phenotypes in an Alzheimer's disease model. Mol Neurodegener. 2023;18(1):93. doi:10.1186/s13024‐023‐00682‐9 38041158 10.1186/s13024-023-00682-9 PMC10693059

[81] Lee YF , Russ AN , Zhao Q , et al. Optogenetic targeting of astrocytes restores slow brain rhythm function and slows Alzheimer's disease pathology. Sci Rep. 2023;13(1):13075. doi:10.1038/s41598‐023‐40402‐3 37567942 10.1038/s41598-023-40402-3 PMC10421876

[82] Kang JE , Lim MM , Bateman RJ , et al. Amyloid‐beta dynamics are regulated by orexin and the sleep‐wake cycle. Science. 2009;326(5955):1005‐1007. doi:10.1126/science.1180962 19779148 10.1126/science.1180962 PMC2789838

[83] Xie L , Kang H , Xu Q , et al. Sleep drives metabolite clearance from the adult brain. Science. 2013;342(6156):373‐377. doi:10.1126/science.1241224 24136970 10.1126/science.1241224 PMC3880190

