# Supplementary material for: Glial cytokine modulation improves sleep and circadian disruption in female SAA knock‐in mice of Alzheimer's‐related pathology
Source: Alzheimers Dement. 2026 Mar 30;22(4):e71314. doi: 10.1002/alz.71314 (PMC13140929; doi:10.1002/alz.71314)
Supplement: Supplementary file 1 — Supporting Information [file ALZ-22-e71314-s001.docx]

**
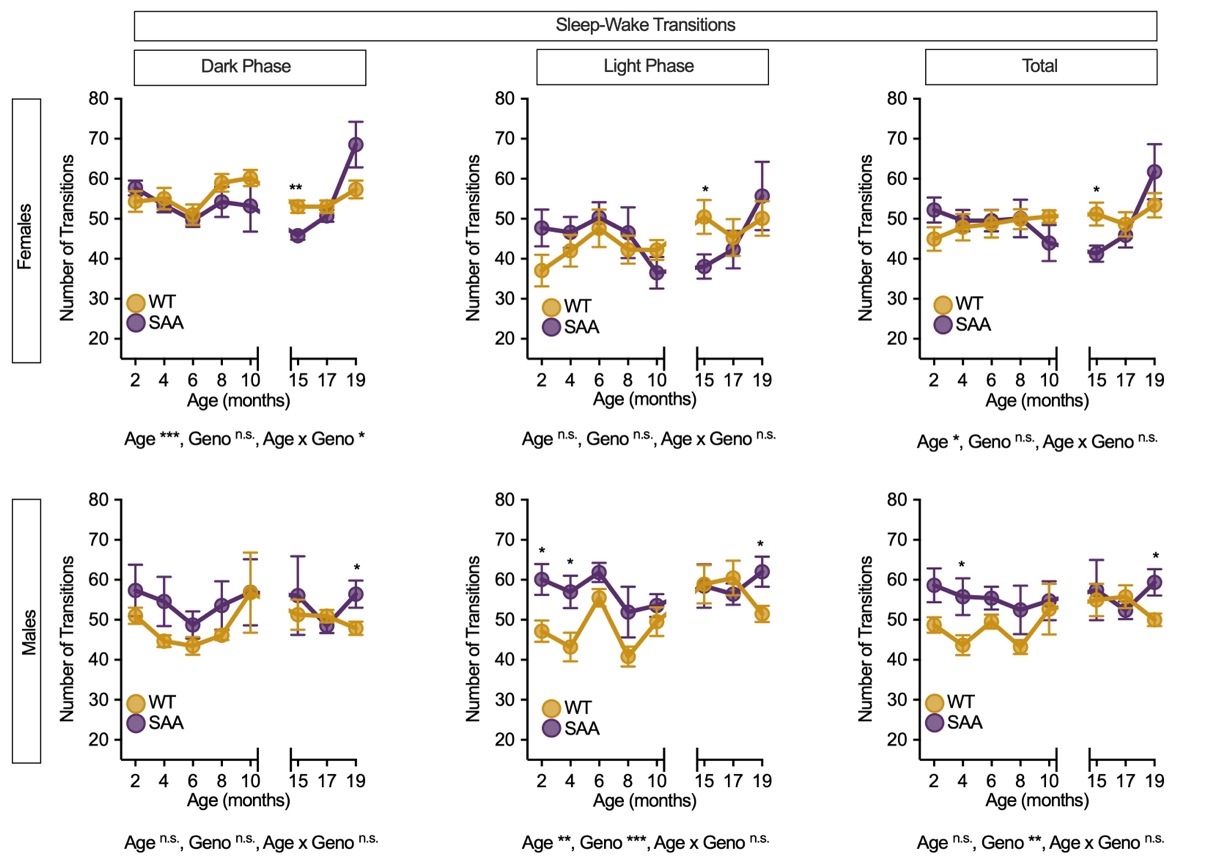
**

**Supplemental Figure 1. Sleep–wake transition frequency across age, phase, and sex.**
Sleep–wake transitions (transitions per hour) were quantified from piezoelectric home-cage recordings across the dark, light, and total 24-hour cycles. The top row shows data from females, and the bottom row shows data from males, with the x-axis indicating age in months. In females, dark-phase transitions showed a significant main effect of age (*F*(7,112)=4.35, *p*=0.0003) and a significant age × genotype interaction (*F*(7,112)=2.12, *p*=0.047), indicating that the pattern of sleep–wake transitions across aging differed between WT and SAA mice. Light-phase transitions did not differ by age or genotype but displayed a modest trend toward reduced flexibility with advancing age (*F*(7,112)=1.59, *p*=0.146). For total 24-hour transitions, a trend for an age effect (*F*(7,112)=2.66, *p*=0.054) suggested an overall reduction in state transitions as females aged, while genotype and interaction terms were nonsignificant (*F*(1,112)=1.61, *p*=0.138). Together, these results indicate that female SAA mice exhibit an early decline in sleep–wake transition frequency—particularly during the dark phase—consistent with reduced flexibility in state regulation. In males, dark-phase transitions remained stable across aging (*F*(7,96)=0.82, *p*=0.569), while light-phase transitions showed a significant effect of age (*F*(7,96)=3.03, *p*=0.0064) and genotype (*F*(1,96)=13.03, *p*=0.0005), with SAA males exhibiting progressively more frequent transitions at later stages. For total transitions, genotype was also significant (*F*(1,96)=8.61, *p*=0.004), suggesting increased sleep–wake instability with age in male SAA mice. These findings reveal a sex-dependent trajectory in sleep–wake regulation: females display early rigidity and reduced transition frequency, while males show later-emerging hyperfragmentation. This temporal divergence mirrors the main-text findings of midlife circadian instability in females and late-onset rhythm disruption in males. Data are presented as mean ± SEM. Asterisks denote significance levels (p < 0.05, **p < 0.01, ***p < 0.001). Statistical outcomes from repeated-measures ANOVA for age, genotype, and age × genotype interaction are reported below each graph. Pairwise comparisons at each age are provided in Supplemental Table 3. Females, WT n = 8, SAA n = 8; males, WT n = 7, SAA n = 7.


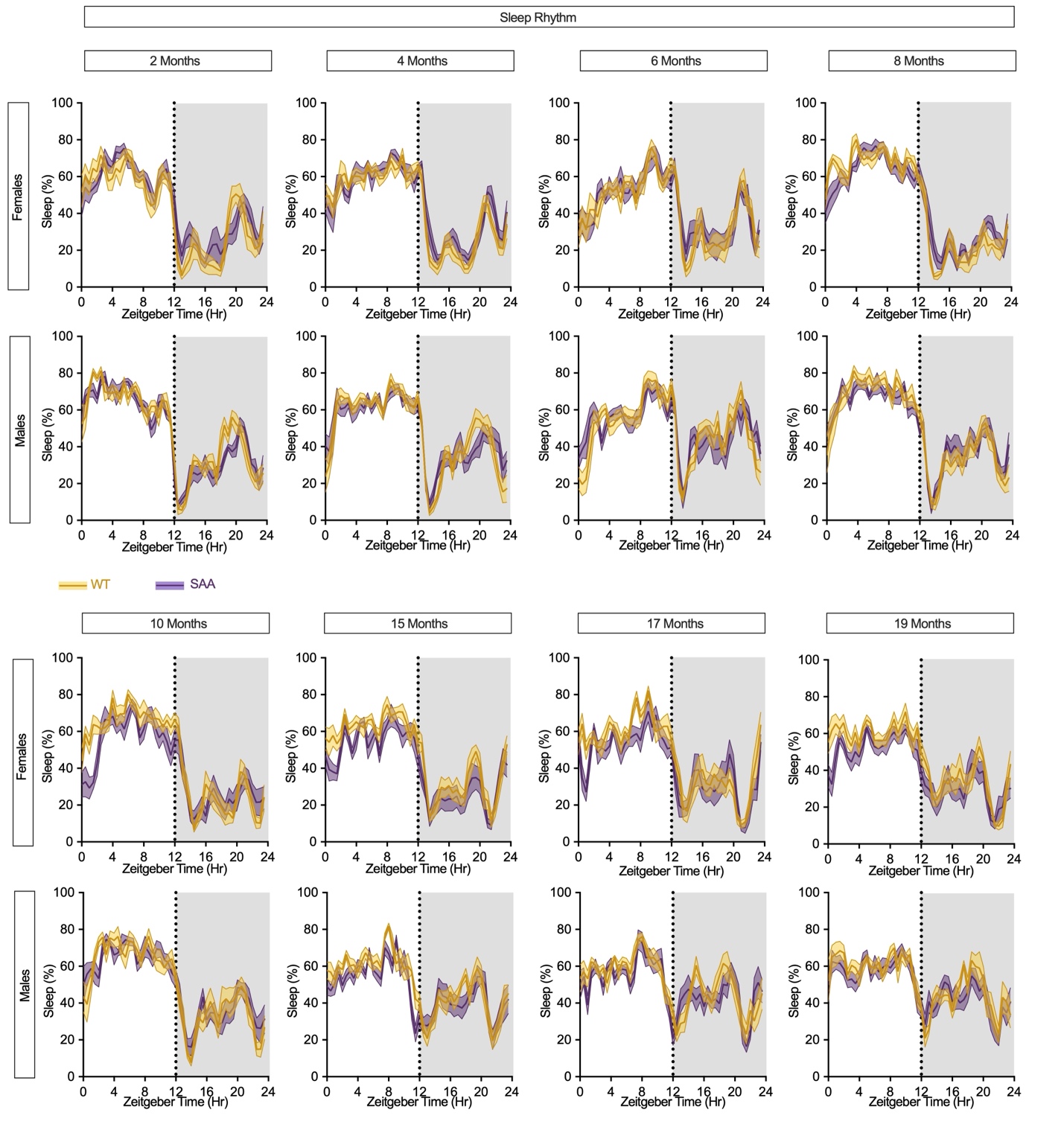


**Supplemental Figure 2. 24 h activity waveforms across aging in hAPP^WT^ KI (WT) and hAPP^SAA^ KI (SAA) mice.** Representative average daily activity profiles recorded with piezoelectric sensors in female (top two rows) and male (bottom two rows) WT (gold) and SAA (purple) mice at 2, 4, 6, 8, 10,15, 17, and 19 months of age. The x-axis indicates Zeitgeber time (ZT, hours relative to lights on at ZT0 and lights off at ZT12), and the shaded background denotes the dark phase. The y-axis represents the percentage of time spent asleep. Data are plotted as mean ± SEM. Females, WT n = 8, SAA n = 8; males, WT n = 7, SAA n = 7.


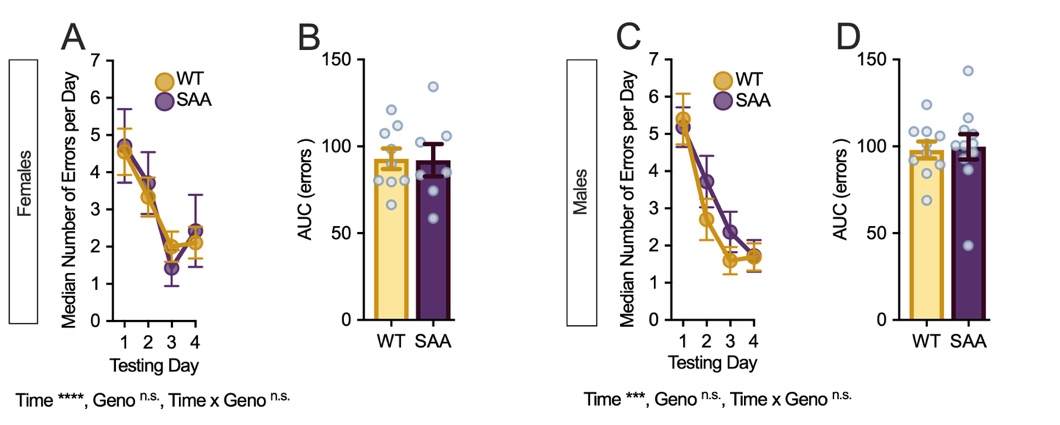


**Supplemental Figure 3. Young hAPP^SAA^ KI mice exhibit normal RAWM acquisition.** To assess spatial learning and memory acquisition, young adult WT and SAA mice (4.28 ± 0.3 months; mean age ± SD ) were tested in the radial arm water maze (RAWM). **(A, C)** Median number of errors per day across the 4-day training protocol for females **(A)** and males **(C)**. Both sexes demonstrated robust learning across days, with significant main effects of time (females: F(3, 59) = 7.73, p = 0.0002; males: F(3, 76) = 8.67, p < 0.0001), but no genotype effects (females: F(1, 59) = 0.02, p = 0.88; males: F(1, 76) = 1.10, p = 0.30) or time × genotype interactions (females: F(3, 59) = 0.27, p = 0.83; males: F(3, 76) = 0.60, p = 0.62). **(B, D)** Area under the curve (AUC) of cumulative errors for females **(B)** and males **(D)** also showed no genotype differences (females: t(10.62) = 0.074, p = 0.94; males: t(17.11) = –0.214, p = 0.83). Each dot represents an individual animal; bars show mean ± SEM. Females: WT (n = 9) and SAA (n = 7); males: WT (n = 10) and SAA (n = 11) mice. Data are presented as mean ± SEM, with individual animal values plotted. Asterisks indicate significance levels for main effects (***p < 0.001, ****p<0.0001). Results indicate that young SAA mice exhibit intact spatial learning and memory, with no early genotype-dependent impairments during RAWM acquisition.


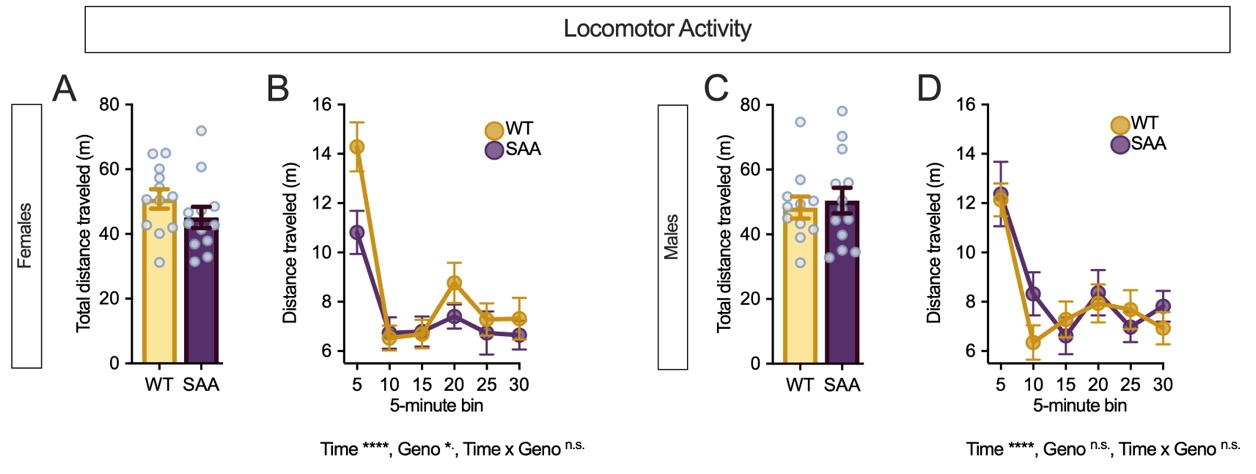


**Supplemental Figure 4. Locomotor activity in hAPP^WT^ KI (WT) and hAPP^SAA^ KI (SAA) .** Locomotor activity was assessed in Cohort 3 mice (8.9 ± 0.4 months) to evaluate general exploratory behavior and overall activity levels. Mice were individually placed in a clear acrylic open-field arena (26.7 cm W × 48.3 cm L × 20.3 cm H) under controlled illumination (200 ± 20 lux) for 30 minutes. Behavior was recorded and analyzed using AnyMaze software (Stoelting Co., Wood Dale, IL), which automatically quantified total distance traveled and distance traveled in 5-minute bins. Following testing, mice were returned to their home cages. In females **(A–B)**, total distance traveled did not differ significantly between WT and SAA mice, although SAA females showed a modest reduction in overall activity compared with WT. Analysis across 5-minute bins revealed robust habituation over time (F(5,132)=18.44, p<0.0001) and a small main effect of genotype (F(1,132)=5.33, p=0.024), with no time × genotype interaction (p=0.13), indicating preserved temporal habituation. In males **(C–D)**, total distance traveled and distance per 5-minute bin were comparable between WT and SAA mice, with significant habituation over time (F(5,132)=12.08, p<0.0001), but no genotype (F(1,132)=0.56, p=0.45) or interaction effects (p=0.58). Females: WT (n = 13) and SAA (n = 12); males: WT (n = 11) and SAA (n = 13) mice. Data are presented as mean ± SEM, with individual animal values plotted. Asterisks indicate significance levels for main effects (*p<0.05, ****p<0.0001). One WT female outlier (total distance traveled = 102.77 m) was excluded from analysis. Results indicate that SAA mice display normal locomotor habituation in both sexes, with only a minor reduction in overall activity in females and no evidence of hyperactivity or altered exploratory behavior.

**
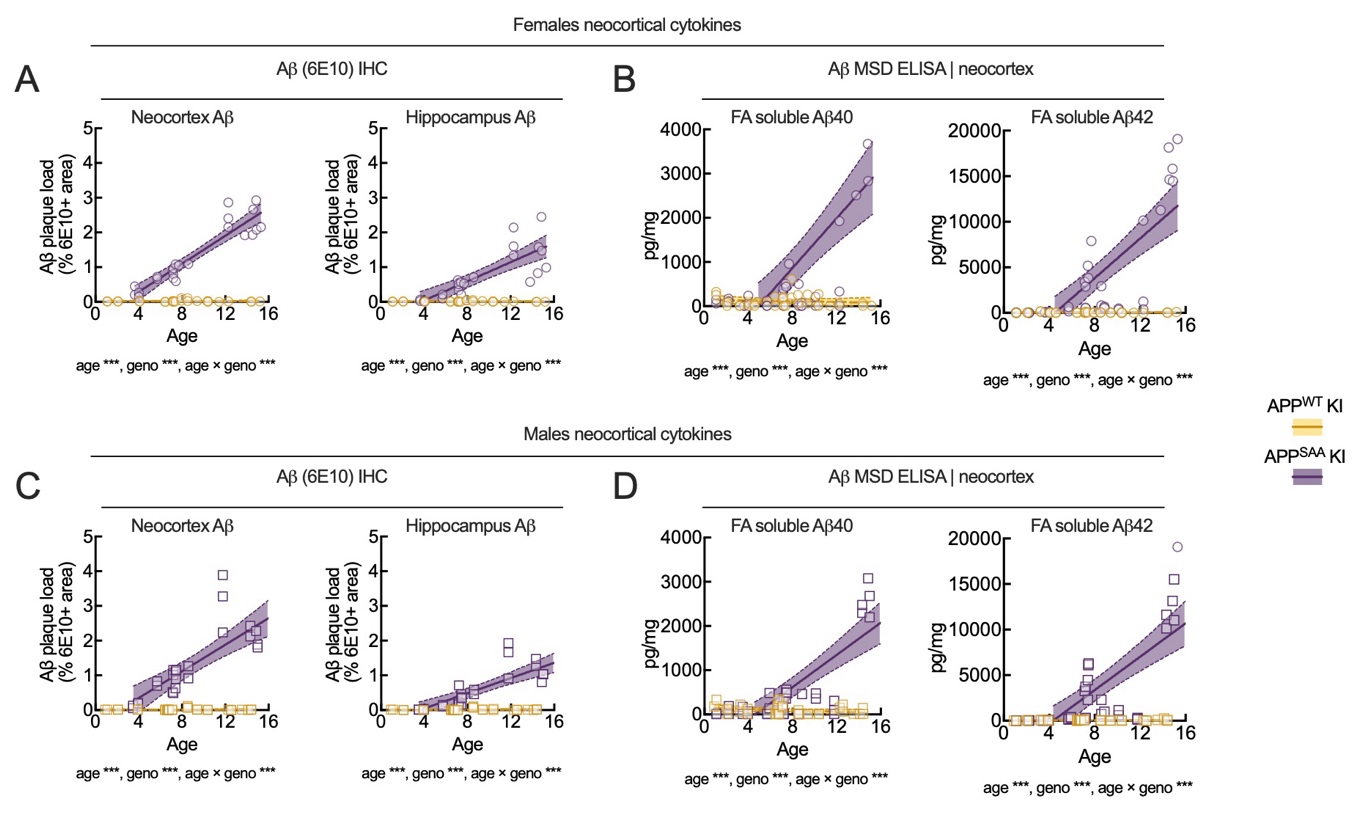
**

**Supplemental Figure 5. Sex-stratified analysis of Aβ accumulation in**  **hAPP**WT  **KI and hAPP**SAA **KI mice. (A)** Aβ plaque load by 6E10 IHC in the neocortex and hippocampus in females. **(B)** Formic acid (FA)-soluble Aβ40 and Aβ42 by MSD ELISA in females. **(C)** Aβ plaque load by 6E10 IHC in the neocortex and hippocampus in males. **(D)** FA-soluble Aβ40 and Aβ42 by MSD ELISA in males. Lines represent mean ± SEM with shaded 95% CIs. Statistical outcomes from two-way ANOVA (age, genotype, age × genotype) performed separately for each sex are shown below each panel. Each point denotes an individual animal. Females: IHC/MSD WT n = 26/31 and SAA n = 23/34; males: WT n = 27/34 and SAA n = 26/35 mice.


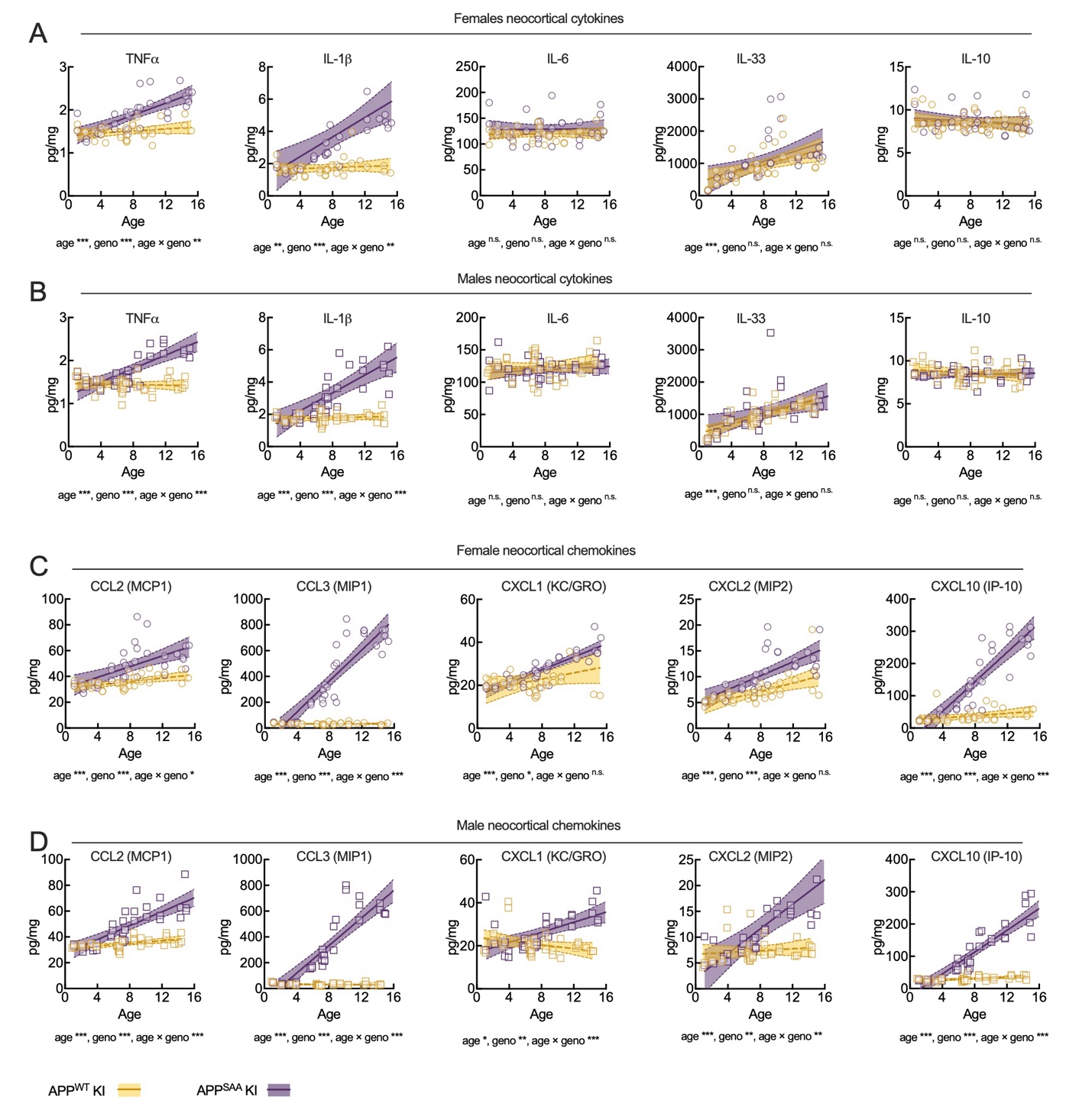


**Supplemental Figure 6. Sex-stratified analysis of neocortical cytokines and chemokines in hAPP**WT **KI and hAPP**SAA **KI mice.** When sex was included in the full factorial model, only CXCL1 (KC/GRO) and CXCL2 (MIP-2) showed significant sex-related effects. CXCL1 showed an age × sex interaction (p=0.007), while CXCL2 showed a genotype × age × sex interaction (p=0.014). Neocortical cytokines in females **(A)** and males **(B)**, and neocortical chemokines in females **(C)** and males **(D)**, with data stratified by sex to visualize these effects. Lines represent mean ± SEM with shaded 95% CIs. Statistical outcomes from two-way ANOVA (age, genotype, age × genotype) performed separately for each sex are shown below each panel. Each point denotes an individual animal. Females: WT n = 31 and SAA n = 34; males: WT n = 34 and SAA n = 34 mice.
