# Supplementary material for: Glial cytokine modulation improves sleep and circadian disruption in female SAA knock‐in mice of Alzheimer's‐related pathology
Source: Alzheimers Dement. 2026 Mar 30;22(4):e71314. doi: 10.1002/alz.71314 (PMC13140929; doi:10.1002/alz.71314)
Supplement: Supplementary file 2 — Supporting Information [file ALZ-22-e71314-s003.docx]

**Supplemental Tables**

**Supplemental Table 1.** Percent sleep across age, phase, and genotype.

|  |  | **Dark Sleep %** | | | **Light Sleep %** | | | **Total Sleep %** | | |
| --- | --- | --- | --- | --- | --- | --- | --- | --- | --- | --- |
|  | **Mo** | **WT** | **SAA** | **P (η²ₚ)** | **WT** | **SAA** | **P (η²ₚ)** | **WT** | **SAA** | **P (η²ₚ)** |
| ***Females*** |  |  |  |  |  |  |  |  |  |  |
|  | 2 | 30.4  (3.5) | 34.1  (3.5) | 0.475  (0.04) | 66.8  (2.2) | 68.5  (2.2) | 0.591  (0.02) | 48.6  (2.3) | 51.3  (2.3) | 0.422  (0.05) |
|  | 4 | 25.4  (1.8) | 30.2  (1.8) | 0.076  (0.21) | 62.0  (1.7) | 62.2  (1.7) | 0.937  (0.00) | 44.0  (1.5) | 46.4  (1.5) | 0.265  (0.09) |
|  | 6 | 34.1  (2.5) | 34.4  (2.5) | 0.936  (0.00) | 56.4  (1.4) | 55.4  (1.4) | 0.605  (0.02) | 44.0  (1.7) | 43.7  (1.7) | 0.905  (0.00) |
|  | 8 | 33.5  (2.9) | 34.1  (2.9) | 0.884  (0.00) | 60.7  (1.7) | 57.9  (1.7) | 0.250  (0.09) | 47.4  (1.9) | 46.2  (1.9) | 0.669  (0.01) |
|  | 10 | 25.1  (2.3) | 26.0  (2.3) | 0.772  (0.01) | 65.4  (2.0) | 57.1  (2.0) | ***0.011**  **(0.38)** | 45.2  (2.0) | 41.6  (2.0) | 0.207  (0.11) |
|  | 15 | 31.4  (2.5) | 26.3  (2.5) | 0.174  (0.13) | 67.4  (1.6) | 57.1  (1.6) | *****<.001**  **(0.60)** | 49.5  (1.8) | 41.9  (1.8) | ****0.009**  **(0.39)** |
|  | 17 | 30.6  (2.8) | 27.9  (2.8) | 0.521  (0.03) | 66.7  (1.4) | 58.6  (1.4) | ****0.001**  **(0.54)** | 48.5  (1.8) | 43.1  (1.8) | 0.051  (0.25) |
|  | 19 | 31.6  (2.5) | 29.0  (2.5) | 0.470  (0.04) | 66.3  (2.2) | 57.4  (2.2) | ***0.014**  **(0.36)** | 49.1  (2.2) | 43.4  (2.2) | 0.092  (0.19) |
| ***Males*** |  |  |  |  |  |  |  |  |  |  |
|  | 2 | 42.9  (2.2) | 44.1  (2.2) | 0.701  (0.01) | 65.4  (2.1) | 65.2  (2.1) | 0.960  (0.00) | 54.1  (1.9) | 54.7  (1.9) | 0.846  (0.00) |
|  | 4 | 35.2  (2.2) | 35.5  (2.2) | 0.937  (0.00) | 62.0  (2.2) | 62.0  (2.2) | 0.998  (0.00) | 48.8  (1.9) | 48.9  (1.9) | 0.963  (0.00) |
|  | 6 | 44.4  (1.8) | 43.3  (1.8) | 0.687  (0.01) | 58.2  (1.4) | 61.1  (1.4) | 0.164  (0.16) | 50.5  (1.3) | 51.2  (1.3) | 0.709  (0.01) |
|  | 8 | 41.5  (1.9) | 41.3  (1.9) | 0.956  (0.00) | 60.0  (2.2) | 61.3  (2.2) | 0.667  (0.02) | 50.9  (1.7) | 51.5  (1.7) | 0.807  (0.01) |
|  | 10 | 32.8  (3.4) | 34.1  (3.4) | 0.786  (0.01) | 65.6  (2.1) | 65.7  (2.1) | 0.985  (0.00) | 49.2  (2.6) | 49.9  (2.6) | 0.851  (0.00) |
|  | 15 | 38.0  (3.1) | 36.6  (3.1) | 0.753  (0.01) | 64.5  (1.7) | 60.0  (1.7) | 0.089  (0.22) | 51.4  (2.1) | 48.4  (2.1) | 0.350  (0.07) |
|  | 17 | 39.8  (1.9) | 38.6  (1.9) | 0.675  (0.02) | 65.8  (1.3) | 63.0  (1.3) | 0.153  (0.16) | 52.7  (1.3) | 50.7  (1.3) | 0.301  (0.09) |
|  | 19 | 40.0  (2.8) | 38.9  (2.8) | 0.792  (0.01) | 65.8  (0.9) | 64.2  (0.9) | 0.215  (0.13) | 53.0  (1.5) | 51.7  (1.5) | 0.527  (0.03) |

Mean on top, (SEM) below. P-value on top, (η²ₚ) below. P-values and partial η² values are shown for genotype comparisons at each age within each phase. Statistical outcomes for main effects of age, genotype, and interactions are reported below each corresponding graph in Figure 1. Significant differences between WT and SAA are highlighted in bold and denoted by asterisks *p < 0.05, **p < 0.01, ***p < 0.001. Sample sizes: n=8 per genotype for females; n=7 per genotype for males at each age. Mo, months of age; WT, hAPP^WT^ KI; SAA, hAPP^SAA^ KI; η²ₚ, partial eta-squared (effect size).

**Supplemental Table 2.** Sleep–wake transitions (per hour) across ages 2–19 months.

|  |  | **Dark Transitions** | | | **Light Transitions** | | | **Total Transitions** | | |
| --- | --- | --- | --- | --- | --- | --- | --- | --- | --- | --- |
|  | **Mo** | **WT** | **SAA** | **P (η²ₚ)** | **WT** | **SAA** | **P (η²ₚ)** | **WT** | **SAA** | **P (η²ₚ)** |
| ***Females*** |  |  |  |  |  |  |  |  |  |  |
|  | 2 | 54.4  (2.2) | 57.7  (2.2) | 0.311  (0.07) | 37.1  (4.3) | 47.8  (4.3) | 0.103  (0.18) | 45.0  (3.0) | 52.2  (3.0) | 0.115  (0.17) |
|  | 4 | 55.0  (2.3) | 53.5  (2.3) | 0.636  (0.02) | 42.1  (3.9) | 46.7  (3.9) | 0.421  (0.05) | 48.0  (3.0) | 49.5  (3.0) | 0.715  (0.01) |
|  | 6 | 51.0  (2.2) | 49.6  (2.2) | 0.656  (0.02) | 47.6  (4.3) | 50.3  (4.3) | 0.669  (0.01) | 48.8  (3.1) | 49.6  (3.1) | 0.856  (0.00) |
|  | 8 | 59.0  (3.1) | 54.2  (3.1) | 0.289  (0.08) | 42.4  (5.1) | 46.5  (5.1) | 0.572  (0.02) | 49.9  (3.8) | 50.1  (3.8) | 0.978  (0.00) |
|  | 10 | 60.2  (4.8) | 53.3  (4.8) | 0.317  (0.07) | 42.3  (3.3) | 36.5  (3.3) | 0.243  (0.10) | 50.6  (3.4) | 44.0  (3.4) | 0.192  (0.12) |
|  | 15 | 53.1  (1.3) | 45.8  (1.3) | ****0.002**  **(0.52)** | 50.5  (3.7) | 38.1  (3.7) | ***0.034**  **(0.28)** | 51.3  (2.5) | 41.3  (2.5) | ***0.014**  **(0.36)** |
|  | 17 | 53.1  (1.5) | 50.7  (1.5) | 0.296  (0.08) | 45.4  (4.7) | 42.3  (4.7) | 0.649  (0.02) | 48.6  (3.0) | 45.9  (3.0) | 0.524  (0.03) |
|  | 19 | 57.4  (4.3) | 68.6  (4.3) | 0.089  (0.19) | 50.1  (6.8) | 55.7  (6.8) | 0.572  (0.02) | 53.4  (5.4) | 61.8  (5.4) | 0.286  (0.08) |
| ***Males*** |  |  |  |  |  |  |  |  |  |  |
|  | 2 | 51.0  (4.8) | 57.4  (4.8) | 0.367  (0.07) | 47.2  (3.4) | 60.1  (3.4) | ***0.019**  **(0.38)** | 48.7  (3.3) | 58.6  (3.3) | 0.055  (0.27) |
|  | 4 | 44.7  (4.5) | 54.6  (4.5) | 0.145  (0.17) | 43.2  (3.8) | 57.0  (3.8) | ***0.025**  **(0.35)** | 43.7  (3.7) | 55.8  (3.7) | ***0.039**  **(0.31)** |
|  | 6 | 43.5  (2.9) | 48.8  (2.9) | 0.219  (0.12) | 55.5  (2.3) | 61.9  (2.3) | 0.079  (0.24) | 49.5  (2.4) | 55.5  (2.4) | 0.102  (0.21) |
|  | 8 | 46.2  (4.4) | 53.6  (4.4) | 0.250  (0.11) | 40.8  (4.9) | 51.9  (4.9) | 0.133  (0.18) | 43.2  (4.5) | 52.5  (4.5) | 0.171  (0.15) |
|  | 10 | 56.9  (9.2) | 56.9  (9.2) | 0.995  (0.00) | 49.6  (3.2) | 53.6  (3.2) | 0.391  (0.06) | 52.7  (5.6) | 54.8  (5.6) | 0.795  (0.01) |
|  | 15 | 51.3  (7.5) | 56.1  (7.5) | 0.659  (0.02) | 58.9  (5.1) | 58.5  (5.1) | 0.958  (0.00) | 55.0  (6.1) | 57.5  (6.1) | 0.779  (0.01) |
|  | 17 | 50.9  (1.7) | 48.6  (1.7) | 0.357  (0.07) | 60.5  (3.6) | 56.5  (3.6) | 0.444  (0.05) | 55.9  (2.6) | 52.4  (2.6) | 0.365  (0.07) |
|  | 19 | 47.9  (2.7) | 56.5  (2.7) | ***0.046**  **(0.29)** | 51.5  (3.0) | 62.1  (3.0) | ***0.028**  **(0.34)** | 50.0  (2.6) | 59.4  (2.6) | ***0.027**  **(0.35)** |

Mean on top, (SEM) below. P-value on top, (η²ₚ) below. Mean ± SEM values for the number of transitions per hour during the dark phase, light phase, and total 24-hour period in female and male mice across ages 2–19 months. P values and partial η² values reflect genotype comparisons (WT vs SAA) at each age. Significant differences between WT and SAA are highlighted in bold and denoted by asterisks *p < 0.05, **p < 0.01. Sample sizes: n=8 per genotype for females; n=7 per genotype for males at each age. Mo, months of age; WT, hAPP^WT^ knock-in; SAA, hAPP^SAA^ knock-in; η²ₚ, partial eta-squared (effect size).

**Supplemental Table 3.** Circadian rhythm parameters across ages 2–19 months.

|  |  | **Amplitude** | | | **MESOR** | | | **Intradaily Var.** | | | **Interdaily Stab.** | | |
| --- | --- | --- | --- | --- | --- | --- | --- | --- | --- | --- | --- | --- | --- |
|  | **Mo** | **WT** | **SAA** | **P (η²ₚ)** | **WT** | **SAA** | **P (η²ₚ)** | **WT** | **SAA** | **P (η²ₚ)** | **WT** | **SAA** | **P (η²ₚ)** |
| ***Females*** |  |  |  |  |  |  |  |  |  |  |  |  |  |
|  | 2 | 1.62  (.09) | 1.60  (.09) | 0.865  (0.00) | 1.61  (.06) | 1.52  (.06) | 0.335  (0.07) | 1.35  (.07) | 1.26  (.07) | 0.341  (0.07) | 0.54  (.03) | 0.52  (.03) | 0.735  (0.01) |
|  | 4 | 2.06  (.08) | 1.92  (.08) | 0.207  (0.11) | 1.57  (.06) | 1.47  (.06) | 0.253  (0.09) | 1.12  (.08) | 1.16  (.08) | 0.766  (0.01) | 0.69  (.02) | 0.63  (.02) | 0.075  (0.21) |
|  | 6 | 1.80  (.11) | 1.62  (.11) | 0.284  (0.08) | 1.59  (.07) | 1.48  (.07) | 0.297  (0.08) | 1.08  (.05) | 1.17  (.05) | 0.203  (0.11) | 0.60  (.03) | 0.55  (.03) | 0.179  (0.13) |
|  | 8 | 2.09  (.10) | 1.77  (.10) | *0.045  (0.26) | 1.51  (.06) | 1.48  (.06) | 0.753  (0.01) | 1.00  (.09) | 1.24  (.09) | 0.075  (0.21) | 0.66  (.03) | 0.58  (.03) | *0.036  (0.28) |
|  | 10 | 1.73  (.07) | 1.49  (.07) | *0.024  (0.32) | 1.59  (.05) | 1.68  (.05) | 0.255  (0.09) | 1.11  (.06) | 1.29  (.06) | 0.056  (0.24) | 0.55  (.03) | 0.49  (.03) | 0.072  (0.21) |
|  | 15 | 1.80  (.11) | 1.61  (.11) | 0.273  (0.09) | 1.40  (.07) | 1.64  (.07) | ***0.022**  **(0.32)** | 1.18  (.11) | 1.53  (.11) | ***0.044**  **(0.26)** | 0.61  (.03) | 0.51  (.03) | ***0.042**  **(0.26)** |
|  | 17 | 1.70  (.13) | 1.51  (.13) | 0.300  (0.08) | 1.41  (.06) | 1.57  (.06) | 0.108  (0.17) | 1.15  (.11) | 1.56  (.11) | ***0.023**  **(0.32)** | 0.61  (.03) | 0.52  (.03) | 0.090  (0.19) |
|  | 19 | 1.52  (.09) | 1.33  (.09) | 0.149  (0.14) | 1.38  (.10) | 1.54  (.10) | 0.296  (0.08) | 1.36  (.08) | 1.64  (.08) | ***0.030**  **(0.30)** | 0.65  (.03) | 0.56  (.03) | ***0.033**  **(0.29)** |
| ***Males*** |  |  |  |  |  |  |  |  |  |  |  |  |  |
|  | 2 | 1.41  (.06) | 1.54  (.06) | 0.136  (0.18) | 1.40  (.05) | 1.40  (.05) | 0.958  (0.00) | 1.19  (.05) | 0.99  (.05) | ***0.014**  **(0.41)** | 0.62  (.02) | 0.64  (.02) | 0.708  (0.01) |
|  | 4 | 1.58  (.08) | 1.46  (.08) | 0.261  (0.10) | 1.46  (.07) | 1.39  (.07) | 0.508  (0.04) | 1.23  (.09) | 1.24  (.09) | 0.974  (0.00) | 0.69  (.04) | 0.69  (.04) | 0.941  (0.00) |
|  | 6 | 1.35  (.08) | 1.16  (.08) | 0.122  (0.19) | 1.29  (.07) | 1.31  (.07) | 0.877  (0.00) | 1.30  (.10) | 1.39  (.10) | 0.539  (0.03) | 0.71  (.04) | 0.62  (.04) | 0.096  (0.21) |
|  | 8 | 1.56  (.11) | 1.30  (.11) | 0.134  (0.18) | 1.36  (.04) | 1.28  (.04) | 0.169  (0.15) | 1.36  (.12) | 1.33  (.12) | 0.868  (0.00) | 0.66  (.04) | 0.62  (.04) | 0.381  (0.06) |
|  | 10 | 1.21  (.12) | 1.14  (.12) | 0.675  (0.02) | 1.41  (.05) | 1.32  (.05) | 0.204  (0.13) | 1.29  (.10) | 1.28  (.10) | 0.939  (0.00) | 0.57  (.04) | 0.52  (.04) | 0.389  (0.06) |
|  | 15 | 1.23  (.08) | 1.04  (.08) | 0.120  (0.19) | 1.32  (.06) | 1.28  (.06) | 0.702  (0.01) | 1.46  (.13) | 1.55  (.13) | 0.595  (0.02) | 0.56  (.04) | 0.53  (.04) | 0.604  (0.02) |
|  | 17 | 1.13  (.10) | 0.91  (.10) | 0.149  (0.17) | 1.25  (.06) | 1.25  (.06) | 0.973  (0.00) | 1.48  (.08) | 1.55  (.08) | 0.574  (0.03) | 0.56  (.03) | 0.49  (.03) | 0.091  (0.22) |
|  | 19 | 1.02  (.10) | 1.02  (.10) | 0.990  (0.00) | 1.29  (.06) | 1.21  (.06) | 0.343  (0.08) | 1.56  (.11) | 1.49  (.11) | 0.682  (0.01) | 0.64  (.04) | 0.62  (.04) | 0.651  (0.02) |

Mean on top, (SEM) below. P-value on top, (η²ₚ) below. Circadian rhythm parameters in female and male mice across ages 2–19 months. P-values and partial η² values reflect genotype comparisons (WT vs SAA) at each age. Statistical outcomes for main effects of age, genotype, and interactions are reported in Supplemental Table 4. Significant differences between WT and SAA are highlighted in bold and denoted by asterisks *p < 0.05. Amplitude and MESOR are expressed in arbitrary units derived from piezoelectric activity signal; IV, intradaily variability; IS, interdaily stability. Sample sizes: n=8 per genotype for females; n=7 per genotype for males at each age. Mo, months of age; WT, hAPP^WT^ knock-in; SAA, hAPP^SAA^ knock-in; η²ₚ, partial eta-squared (effect size).

**Supplemental Table 4.** Full factorial analysis (Genotype × Sex × Age).

| **Endpoint** | **Geno** | **Sex** | **Age** | **G × S** | **G × A** | **S × A** | **G×S×A** |
| --- | --- | --- | --- | --- | --- | --- | --- |
| Sleep (Dark) | 0.892 | <0.001  (0.32) | <0.001  (0.18) | 0.885 | 0.703 | 0.970 | 0.972 |
| Sleep (Light) | <0.001  (0.08) | 0.020  (0.03) | <0.001  (0.22) | 0.002  (0.05) | 0.003  (0.10) | 0.143 | 0.472 |
| Sleep (Total) | 0.038  (0.02) | <0.001  (0.23) | 0.001  (0.11) | 0.159 | 0.088 | 0.896 | 0.773 |
| Amplitude | <0.001  (0.08) | <0.001  (0.45) | <0.001  (0.32) | 0.316 | 0.378 | 0.046  (0.07) | 0.980 |
| MESOR | 0.928 | <0.001  (0.27) | 0.013  (0.08) | 0.081 | 0.383 | 0.686 | 0.210 |
| Intradaily var. | 0.005  (0.04) | 0.004  (0.04) | <0.001  (0.21) | 0.003  (0.04) | 0.103 | 0.026  (0.07) | 0.793 |
| Interdaily stab. | <0.001  (0.10) | 0.008  (0.03) | <0.001  (0.26) | 0.154 | 0.668 | 0.022  (0.08) | 0.944 |
| Nesting | <0.001  (0.16) | 0.160 | <0.001  (0.37) | 0.104 | 0.160 | 0.453 | 0.946 |

P-value on top, (η²ₚ) below for significant effects. G, Genotype; S, Sex; A, Age. η²ₚ, partial eta-squared (effect size)

**Supplemental Table 5.** Age, genotype, and interaction effects on amyloid and inflammatory markers.

|  | **Age** | | **Genotype** | | **Age × Genotype** | |
| --- | --- | --- | --- | --- | --- | --- |
| **Measure** | **F** | **P (η²ₚ)** | **F** | **P (η²ₚ)** | **F** | **P (η²ₚ)** |
| ***Amyloid Pathology*** |  |  |  |  |  |  |
| Neocortex (IHC) | 130.38 | <0.001  (0.56) | 93.59 | <0.001  (0.48) | 92.47 | <0.001  (0.48) |
| Hippocampus (IHC) | 320.88 | <0.001  (0.84) | 140.26 | <0.001  (0.70) | 139.65 | <0.001  (0.70) |
| Aβ40 (ELISA) | 54.15 | <0.001  (0.30) | 24.35 | <0.001  (0.16) | 59.98 | <0.001  (0.32) |
| Aβ42 (ELISA) | 73.57 | <0.001  (0.36) | 55.10 | <0.001  (0.30) | 73.24 | <0.001  (0.36) |
| ***Cytokines*** |  |  |  |  |  |  |
| TNF-α | 35.89 | <0.001  (0.22) | 66.38 | <0.001  (0.34) | 27.66 | <0.001  (0.18) |
| IL-1β | 55.69 | <0.001  (0.30) | 121.95 | <0.001  (0.49) | 33.52 | <0.001  (0.21) |
| IL-6 | 0.02 | 0.90  (0.00) | 0.73 | 0.39  (0.01) | 0.04 | 0.84  (0.00) |
| IL-10 | 0.39 | 0.53  (0.00) | 1.67 | 0.20  (0.01) | 0.01 | 0.94  (0.00) |
| IL-33 | 1.82 | 0.18  (0.01) | 0.32 | 0.57  (0.00) | 0.55 | 0.46  (0.00) |
| ***Chemokines*** |  |  |  |  |  |  |
| CCL2 | 60.17 | <0.001  (0.32) | 31.99 | <0.001  (0.20) | 2.79 | 0.10  (0.02) |
| CCL3 | 110.36 | <0.001  (0.46) | 76.83 | <0.001  (0.37) | 40.99 | <0.001  (0.24) |
| CXCL1 | 16.64 | <0.001  (0.11) | 149.52 | <0.001  (0.54) | 2.26 | 0.14  (0.02) |
| CXCL2 | 27.97 | <0.001  (0.18) | 456.74 | <0.001  (0.78) | 6.71 | 0.011  (0.05) |
| CXCL10 | 69.13 | <0.001  (0.35) | 148.81 | <0.001  (0.54) | 3.25 | 0.07  (0.03) |

Two-way ANOVAs (Age × Genotype). F-value shown; P-value on top, (η²ₚ) below. Degrees of freedom: df = (1, 102) for neocortex IHC; df = (1, 101) for hippocampus IHC; df = (1, 129) for Aβ ELISA (MSD) and cytokine/chemokine (MSD) measures in the neocortex. Two-way ANOVAs were conducted to evaluate main effects of age, genotype, and age × genotype interactions on amyloid load and neocortical cytokine/chemokine expression in SAA and WT mice. Partial η² values represent effect size estimates. IHC, immunohistochemistry; MSD, Meso Scale Discovery; η²ₚ, partial eta-squared (effect size).

**Supplemental Table 6.** Two-way ANOVA results for MW151 treatment effects on sleep and circadian parameters in hAPP^SAA^ mice.

|  | **Treatment** | | **Sex** | | **Treatment × Sex** | |
| --- | --- | --- | --- | --- | --- | --- |
| **Endpoint** | **F** | **p (η²ₚ)** | **F** | **p (η²ₚ)** | **F** | **p (η²ₚ)** |
| ***Sleep*** | | | | | | |
| Sleep %, light phase | 8.03 | 0.006  (0.109) | 2.64 | 0.109  (0.039) | 0.00 | 0.991  (0.000) |
| Sleep %, dark phase | 0.14 | 0.709  (0.002) | 0.29 | 0.593  (0.004) | 2.23 | 0.140  (0.033) |
| Sleep %, 24-hour | 3.33 | 0.072  (0.048) | 4.49 | 0.038  (0.064) | 0.98 | 0.325  (0.015) |
| ***Circadian rhythm*** | | | | | | |
| Amplitude | 0.11 | 0.745  (0.002) | 1.25 | 0.267  (0.019) | 0.92 | 0.341  (0.014) |
| MESOR | 5.79 | 0.019  (0.081) | 5.02 | 0.028  (0.071) | 0.17 | 0.685  (0.003) |
| IV | 0.49 | 0.487  (0.007) | 0.12 | 0.732  (0.002) | 0.66 | 0.420  (0.010) |

Two-way ANOVAs (Treatment × Sex) for percent change from baseline to Week 5 in sleep and circadian endpoints. All F-statistics have df = (1, 66). P-value on top, (η²ₚ) below. IV, intradaily variability; MESOR, midline estimating statistic of rhythm; η²ₚ, partial eta-squared (effect size).

**Supplemental Table 7.** Summary of treatment and sex effects on Aβ levels and plaque morphology following MW151 administration.

|  | **Treatment** | | **Sex** | | **Treatment × Sex** | |
| --- | --- | --- | --- | --- | --- | --- |
| **Endpoint** | **F** | **P (η²ₚ)** | **F** | **P (η²ₚ)** | **F** | **P (η²ₚ)** |
| ***Aβ ELISA – Cortical Fractions (fmol/mg protein)*** | | | | | | |
| Aβ, PBS-soluble | 1.21 | 0.276  (0.018) | 7.55 | 0.008  (0.103) | 2.12 | 0.15  (0.031) |
| Aβ, detergent-soluble (T-PER) | 1.56 | 0.217  (0.023) | 1.74 | 0.191  (0.026) | 0.24 | 0.625  (0.004) |
| Aβ, formic acid-soluble | 2.52 | 0.117  (0.037) | 0.5 | 0.482  (0.008) | 0.53 | 0.468  (0.008) |
| ***Aβ Immunohistochemistry – Plaque Density (plaques per mm²)*** | | | | | | |
| Aβ plaques, small (<250 μm²), neocortex | 0.36 | 0.551  (0.005) | 10.66 | 0.002  (0.139) | 0.42 | 0.519  (0.006) |
| Aβ plaques, medium (250–2500 μm²), neocortex | 0.01 | 0.935  (0.00) | 9.01 | 0.004  (0.12) | 0.01 | 0.927  (0.00) |
| Aβ plaques, large (>2500 μm²), neocortex | 0.73 | 0.394  (0.011) | 8.01 | 0.006  (0.108) | 0.73 | 0.396  (0.011) |
| Aβ plaques, small (<250 μm²), hippocampus | 0.04 | 0.84  (0.001) | 0.15 | 0.696  (0.002) | 0.65 | 0.423  (0.01) |
| Aβ plaques, medium (250–2500 μm²), hippocampus | 0.08 | 0.774  (0.001) | 5.01 | 0.029  (0.071) | 0.54 | 0.464  (0.008) |
| Aβ plaques, large (>2500 μm²), hippocampus | 1.92 | 0.171  (0.028) | 7.46 | 0.008  (0.102) | 0.64 | 0.426  (0.01) |

Two-way ANOVAs were performed to examine main effects of treatment (vehicle vs. MW151), sex, and treatment × sex interactions on cortical Aβ levels (ELISA) and plaque morphology (IHC) in 15-month-old SAA mice. MW151 treatment did not significantly alter soluble, detergent-soluble, or formic acid-soluble Aβ fractions, nor did it affect plaque density across size categories in either the neocortex or hippocampus. Significant main effects of sex were detected for several Aβ endpoints, reflecting modestly higher Aβ burden in females. Partial η² values represent effect size estimates. Two-way ANOVAs (Treatment × Sex). F-value shown; P-value on top, (η²ₚ) below. All F-statistics have df = (1, 66). η²ₚ, partial eta-squared (effect size).

**Supplemental Table 8.** Summary of treatment and sex effects on glial gene expression following MW151 administration.

|  | **Treatment** | | **Sex** | | **Treatment × Sex** | |
| --- | --- | --- | --- | --- | --- | --- |
| **Gene** | **F** | **P (η²ₚ)** | **F** | **P (η²ₚ)** | **F** | **P (η²ₚ)** |
| *CD68* | 0.57 | 0.454  (0.009) | 11.62 | 0.001  (0.15) | 0.29 | 0.594  (0.004) |
| *Clec7a (Dectin-1)* | 0.4 | 0.529  (0.006) | 6.22 | 0.015  (0.086) | 1.08 | 0.303  (0.016) |
| *GFAP* | 0.06 | 0.809  (0.001) | 11.3 | 0.001  (0.146) | 0.8 | 0.374  (0.012) |
| *LCN2 (lipocalin-2)* | 0.51 | 0.477  (0.008) | 0.18 | 0.676  (0.003) | 3.03 | 0.086  (0.044) |
| *PTX3 (pentraxin-3)* | 2.15 | 0.147  (0.032) | 8.91 | 0.004  (0.119) | 2.37 | 0.128  (0.035) |
| *TREM2* | 1.08 | 0.303  (0.016) | 9.03 | 0.004  (0.12) | 0.32 | 0.576  (0.005) |

Two-way ANOVAs (Treatment × Sex). F-value shown; P-value on top, (η²ₚ) below. All F-statistics have df = (1, 66). Data expressed as fold change relative to vehicle. η²ₚ, partial eta-squared (effect size).

**Supplemental Table 9.** Summary of treatment and sex effects on glial immunohistochemistry following MW151 administration.

|  | **Treatment** | | **Sex** | | **Treatment × Sex** | |
| --- | --- | --- | --- | --- | --- | --- |
| **Endpoint** | **F** | **P (η²ₚ)** | **F** | **P (η²ₚ)** | **F** | **P (η²ₚ)** |
| ***Percent Area*** | | | | | | |
| GFAP, Ctx | 0.87 | 0.353  (0.013) | 0.67 | 0.417  (0.01) | 0.04 | 0.844  (0.001) |
| GFAP, Hpc | 1.34 | 0.252  (0.02) | 3.22 | 0.077  (0.048) | 0.47 | 0.494  (0.007) |
| Dectin-1, Ctx | 0.03 | 0.875  (0.00) | 0.06 | 0.813  (0.001) | 0.49 | 0.484  (0.007) |
| Dectin-1, Hpc | 0.06 | 0.815  (0.001) | 0.34 | 0.561  (0.005) | 0.81 | 0.37  (0.012) |
| CD45, Ctx | 0.44 | 0.509  (0.009) | 2.46 | 0.123  (0.047) | 0.59 | 0.445  (0.012) |
| CD45, Hpc | 0.0 | 0.978  (0.00) | 0.77 | 0.383  (0.015) | 0.29 | 0.596  (0.006) |
| ***Cluster Density (clusters per mm²)*** | | | | | | |
| Dectin-1, small (<200 μm²), Ctx | 0.12 | 0.731  (0.002) | 1.8 | 0.184  (0.027) | 0.31 | 0.577  (0.005) |
| Dectin-1, medium (200–1000 μm²), Ctx | 1.09 | 0.3  (0.016) | 2.75 | 0.102  (0.04) | 0.55 | 0.46  (0.008) |
| Dectin-1, large (>1000 μm²), Ctx | 0.08 | 0.774  (0.001) | 0.13 | 0.719  (0.002) | 0.18 | 0.674  (0.003) |
| Dectin-1, small (<200 μm²), Hpc | 0.04 | 0.852  (0.001) | 0.43 | 0.514  (0.006) | 1.05 | 0.31  (0.016) |
| Dectin-1, medium (200–1000 μm²), Hpc | 1.22 | 0.274  (0.018) | 0.89 | 0.348  (0.013) | 0.0 | 0.952  (0.00) |
| Dectin-1, large (>1000 μm²), Hpc | 0.66 | 0.419  (0.01) | 0.05 | 0.816  (0.001) | 0.37 | 0.548  (0.006) |
| CD45, small (<200 μm²), Ctx | 0.05 | 0.819  (0.001) | 0.22 | 0.64  (0.004) | 0.05 | 0.832  (0.001) |
| CD45, medium (200–1000 μm²), Ctx | 0.01 | 0.935  (0.00) | 6.21 | 0.016  (0.111) | 0.6 | 0.444  (0.012) |
| CD45, large (>1000 μm²), Ctx | 0.74 | 0.394  (0.015) | 1.68 | 0.201  (0.032) | 0.63 | 0.433  (0.012) |
| CD45, small (<200 μm²), Hpc | 0.56 | 0.459  (0.011) | 0.9 | 0.347  (0.018) | 0.49 | 0.486  (0.01) |
| CD45, medium (200–1000 μm²), Hpc | 0.16 | 0.688  (0.003) | 1.44 | 0.237  (0.028) | 0.58 | 0.451  (0.011) |
| CD45, large (>1000 μm²), Hpc | 0.45 | 0.508  (0.009) | 0.28 | 0.596  (0.006) | 1.08 | 0.304  (0.021) |

Two-way ANOVAs (Treatment × Sex). F-value shown; P-value on top, (η²ₚ) below. df = (1, 64) for GFAP; df = (1, 66) for Dectin-1; df = (1, 50) for CD45. Ctx, neocortex; Hpc, hippocampus. η²ₚ, partial eta-squared (effect size).

**Supplemental Table 10.** Sleep parameters in the MW151 treatment study by genotype, treatment, and sex at baseline and week 5.

| **Group** | **Sex** | **Dark Phase** | **Light Phase** | **Total** | **n** |
| --- | --- | --- | --- | --- | --- |
| ***Baseline*** | | | | | |
| SAA + MW151 | F | 28.6  (5.0) | 63.4  (3.8) | 46.0  (2.9) | 17 |
|  | M | 32.5  (10.2) | 60.9  (8.0) | 46.7  (8.8) | 19 |
| SAA + Vehicle | F | 26.2  (6.6) | 63.3  (5.5) | 44.8  (5.4) | 17 |
|  | M | 33.5  (9.0) | 63.8  (7.4) | 48.7  (7.4) | 19 |
| WT + MW151 | F | 29.6  (11.7) | 66.2  (8.2) | 47.9  (9.7) | 8 |
|  | M | 33.2  (8.2) | 65.9  (7.6) | 49.5  (7.5) | 8 |
| WT + Vehicle | F | 29.9  (7.8) | 62.4  (6.6) | 46.2  (5.7) | 8 |
|  | M | 37.3  (6.0) | 66.6  (5.4) | 52.0  (4.6) | 8 |
| ***Week 5*** | | | | | |
| SAA + MW151 | F | 34.5  (5.0) | 61.0  (4.8) | 47.2  (3.4) | 17 |
|  | M | 40.6  (8.3) | 59.6  (6.3) | 50.7  (6.7) | 19 |
| SAA + Vehicle | F | 32.7  (6.5) | 56.6  (6.0) | 44.0  (5.1) | 17 |
|  | M | 38.7  (7.6) | 59.6  (6.2) | 49.8  (6.3) | 19 |
| WT + MW151 | F | 32.6  (11.0) | 60.0  (6.0) | 46.0  (7.8) | 8 |
|  | M | 31.1  (16.2) | 52.3  (21.6) | 42.3  (18.4) | 8 |
| WT + Vehicle | F | 35.9  (11.0) | 59.4  (4.6) | 47.7  (7.1) | 8 |
|  | M | 38.4  (11.2) | 62.5  (5.4) | 51.1  (8.1) | 8 |

Mean on top, (SD) below. SAA, hAPP^SAA^ KI; WT, hAPP^WT^ KI.

**Supplemental Table 11.** Circadian rhythm parameters in the MW151 treatment study by genotype, treatment, and sex at baseline and week 5.

| **Group** | **Sex** | **Amplitude** | **MESOR** | **IV** | **IS** | **n** |
| --- | --- | --- | --- | --- | --- | --- |
| ***Baseline*** | | | | | | |
| SAA + MW151 | F | 1.39  (0.40) | 1.49  (0.14) | 1.27  (0.24) | 0.61  (0.13) | 17 |
|  | M | 0.96  (0.28) | 1.39  (0.20) | 1.36  (0.31) | 0.58  (0.13) | 19 |
| SAA + Vehicle | F | 1.48  (0.31) | 1.48  (0.20) | 1.26  (0.23) | 0.62  (0.12) | 17 |
|  | M | 0.99  (0.32) | 1.35  (0.21) | 1.38  (0.39) | 0.59  (0.12) | 19 |
| WT + MW151 | F | 1.41  (0.50) | 1.26  (0.27) | 1.06  (0.20) | 0.58  (0.21) | 8 |
|  | M | 1.14  (0.23) | 1.33  (0.25) | 1.29  (0.41) | 0.51  (0.14) | 8 |
| WT + Vehicle | F | 1.28  (0.44) | 1.34  (0.23) | 1.28  (0.20) | 0.55  (0.15) | 8 |
|  | M | 0.99  (0.25) | 1.20  (0.13) | 1.28  (0.25) | 0.48  (0.09) | 8 |
| ***Week 5*** | | | | | | |
| SAA + MW151 | F | 1.19  (0.39) | 1.33  (0.17) | 1.24  (0.28) | 0.53  (0.11) | 17 |
|  | M | 0.81  (0.27) | 1.20  (0.23) | 1.38  (0.26) | 0.52  (0.11) | 19 |
| SAA + Vehicle | F | 1.07  (0.28) | 1.44  (0.24) | 1.22  (0.23) | 0.54  (0.12) | 17 |
|  | M | 0.86  (0.26) | 1.21  (0.24) | 1.28  (0.27) | 0.50  (0.13) | 19 |
| WT + MW151 | F | 1.10  (0.34) | 1.32  (0.28) | 1.24  (0.33) | 0.51  (0.10) | 8 |
|  | M | 0.80  (0.46) | 1.05  (0.48) | 1.41  (0.43) | 0.46  (0.12) | 8 |
| WT + Vehicle | F | 1.11  (0.42) | 1.33  (0.15) | 1.39  (0.37) | 0.58  (0.09) | 8 |
|  | M | 0.99  (0.31) | 1.21  (0.16) | 1.25  (0.31) | 0.50  (0.07) | 8 |

Mean on top, (SD) below. SAA, hAPP^SAA^ KI; WT, hAPP^WT^ KI; IV, intradaily variability; IS, interdaily stability; MESOR, midline estimating statistic of rhythm.

**Supplemental Table 12.** Neocortical cytokine and chemokine levels in the MW151 treatment study by genotype, treatment, and sex.

| ***Cytokines (pg/mg protein)*** | | | | | | | |
| --- | --- | --- | --- | --- | --- | --- | --- |
| **Group** | **Sex** | **IL-10** | **IL-1β** | **IL-6** | **TNF-α** | **IL-33** | **n** |
| SAA + MW151 | F | 5.88  (0.84) | 3.29  (0.55) | 32.0  (7.1) | 1.50  (0.25) | 1086  (226) | 13 |
|  | M | 6.51  (1.81) | 2.36  (0.39) | 29.2  (4.5) | 1.42  (0.25) | 1021  (197) | 16 |
| SAA + Vehicle | F | 6.67  (1.30) | 3.49  (0.56) | 34.0  (8.9) | 1.63  (0.31) | 1086  (161) | 14 |
|  | M | 6.59  (1.66) | 2.62  (0.69) | 32.9  (7.9) | 1.69  (0.45) | 1180  (332) | 15 |
| WT + MW151 | F | 6.89  (1.46) | 1.28  (0.22) | 32.9  (4.6) | 1.04  (0.25) | 742  (140) | 6 |
|  | M | 8.16  (0.97) | 1.27  (0.37) | 49.7  (12.6) | 0.94  (0.07) | 849  (76.0) | 5 |
| WT + Vehicle | F | 7.92  (1.32) | 1.25  (0.19) | 35.1  (4.4) | 0.89  (0.22) | 765  (100) | 6 |
|  | M | 8.58  (1.46) | 1.21  (0.20) | 37.1  (6.0) | 0.92  (0.12) | 926  (231) | 6 |
| ***Chemokines (pg/mg protein)*** | | | | | | | |
| **Group** | **Sex** | **CXCL1** | **CXCL2** | **CXCL10** | **CCL2** | **CCL3** | **n** |
| SAA + MW151 | F | 58.7  (8.2) | 7.97  (1.76) | 297  (85.0) | 64.7  (28.5) | 542  (150) | 13 |
|  | M | 60.8  (15.3) | 8.87  (1.66) | 227  (48.0) | 65.9  (12.3) | 460  (98.0) | 16 |
| SAA + Vehicle | F | 60.4  (13.9) | 8.82  (1.49) | 300  (65.0) | 62.9  (7.2) | 519  (126) | 14 |
|  | M | 60.3  (10.5) | 10.4  (5.50) | 275  (110) | 79.5  (21.1) | 558  (153) | 15 |
| WT + MW151 | F | 33.0  (7.9) | 5.94  (0.59) | 55.2  (16.9) | 47.7  (13.6) | 33.7  (10) | 6 |
|  | M | 86.1  (121) | 13.8  (17.3) | 50.2  (13.7) | 44.8  (3.5) | 27.9  (7) | 5 |
| WT + Vehicle | F | 26.5  (4.9) | 5.28  (0.81) | 69.5  (48.0) | 39.0  (5.8) | 34.0  (8) | 6 |
|  | M | 32.1  (7.9) | 5.99  (1.17) | 40.1  (9) | 41.9  (4.7) | 31.7  (5) | 6 |

Mean on top, (SD) below. Cytokine and chemokine concentrations measured by MSD in neocortical tissue from 15-month-old mice. SAA, hAPP^SAA^ KI; WT, hAPP^WT^ KI; MSD, Meso Scale Discovery.

**Supplemental Table 13.** Neocortical Aβ levels in the MW151 treatment study by genotype, treatment, and sex.

| ***Aβ ELISA (fmol/mg protein)*** | | | | | | |
| --- | --- | --- | --- | --- | --- | --- |
| **Group** | **Sex** | **PBS-soluble** | **T-PER** | **Formic acid** |  | **n** |
| SAA + MW151 | F | 45.2  (12.7) | 24.2  (6.5) | 25234  (21434) |  | 16 |
|  | M | 36.2  (7.4) | 23.0  (5.1) | 25354  (20439) |  | 18 |
| SAA + Vehicle | F | 39.7  (8.5) | 23.1  (7.1) | 37272  (25207) |  | 17 |
|  | M | 36.9  (6.8) | 20.6  (4.5) | 29807  (19491) |  | 19 |
| WT + MW151 | F | 37.7  (9.1) | 25.1  (5.6) | 3336  (1703) |  | 8 |
|  | M | 40.8  (12.0) | 22.6  (3.4) | 2760  (2136) |  | 7 |
| WT + Vehicle | F | 53.0  (20.3) | 24.5  (3.1) | 2136  (1968) |  | 8 |
|  | M | 43.9  (18.7) | 26.0  (3.7) | 2227  (1065) |  | 8 |
| ***Aβ IHC — Neocortex*** | | | | | | |
| **Group** | **Sex** | **% Area** | **<250 μm²** | **250–2500 μm²** | **>2500 μm²** | **n** |
| SAA + MW151 | F | 89.5  (7.9) | 3.38  (0.657) | 23.7  (4.45) | 1.61  (0.521) | 16 |
|  | M | 90.9  (4.9) | 2.88  (0.514) | 20.8  (4.72) | 1.36  (0.616) | 18 |
| SAA + Vehicle | F | 90.6  (6.0) | 3.22  (0.461) | 23.7  (3.86) | 1.83  (0.414) | 17 |
|  | M | 89.3  (7.7) | 2.88  (0.494) | 20.6  (3.76) | 1.36  (0.575) | 19 |
| WT + MW151 | F | 86.3  (6.8) | 0.159  (0.099) | 0.377  (0.260) | 0.042  (0.044) | 8 |
|  | M | 88.4  (3.6) | 0.150  (0.130) | 0.502  (0.467) | 0.018  (0.022) | 7 |
| WT + Vehicle | F | 87.6  (5.1) | 0.235  (0.115) | 0.436  (0.270) | 0.015  (0.018) | 8 |
|  | M | 91.5  (5.2) | 0.206  (0.100) | 0.351  (0.125) | 0.019  (0.019) | 8 |

Mean on top, (SD) below. Aβ levels measured by MSD ELISA in cortical fractions and plaque burden quantified by immunohistochemistry in neocortex of 15-month-old mice. PBS, phosphate-buffered saline (soluble fraction); T-PER, tissue protein extraction reagent (detergent-soluble fraction); Formic acid, formic acid-soluble fraction (insoluble/aggregated). Plaque density expressed as plaques per mm² × 10⁻³. SAA, hAPP^SAA^ KI; WT, hAPP^WT^ KI; MSD, Meso Scale Discovery; IHC, immunohistochemistry.

**Supplemental Table 14.** Neocortical glial markers in the MW151 treatment study by genotype, treatment, and sex.

| ***Gene expression, qPCR (fold change)*** | | | | | |
| --- | --- | --- | --- | --- | --- |
| **Group** | **Sex** | ***Gfap*** | ***Lcn2*** | ***Ptx3*** | **n** |
| SAA + MW151 | F | 9.17  (2.91) | 4.39  (5.06) | 5.80  (1.65) | 16 |
|  | M | 6.57  (1.65) | 2.75  (2.20) | 3.77  (2.46) | 18 |
| SAA + Vehicle | F | 8.47  (2.94) | 2.53  (0.90) | 4.45  (1.37) | 17 |
|  | M | 6.97  (2.54) | 3.53  (3.18) | 3.80  (1.79) | 19 |
| WT + MW151 | F | 1.26  (0.39) | 1.65  (1.65) | 1.22  (0.67) | 8 |
|  | M | 0.98  (0.27) | 9.82  (23.9) | 1.17  (1.18) | 7 |
| WT + Vehicle | F | 1.16  (0.31) | 1.55  (1.17) | 1.29  (0.68) | 8 |
|  | M | 0.89  (0.12) | 0.94  (0.63) | 1.04  (0.55) | 8 |
| ***Immunohistochemistry (% area)*** | | | | | |
| **Group** | **Sex** | **GFAP** | **CD45** | **Dectin-1** | **n** |
| SAA + MW151 | F | 10.7  (2.95) | 5.50  (1.45) | 1.08  (0.42) | 11–16 |
|  | M | 10.3  (2.35) | 5.21  (1.10) | 1.12  (0.45) | 15–18 |
| SAA + Vehicle | F | 11.4  (2.83) | 6.03  (1.64) | 1.16  (0.36) | 14–17 |
|  | M | 10.8  (2.90) | 5.17  (1.18) | 1.07  (0.35) | 14–19 |
| WT + MW151 | F | 0.69  (0.31) | 0.087  (0.033) | 0.0063  (0.0020) | 5–8 |
|  | M | 0.48  (0.21) | 0.058  (0.013) | 0.0049  (0.0029) | 5–7 |
| WT + Vehicle | F | 0.69  (0.23) | 0.069  (0.025) | 0.0055  (0.0031) | 6–8 |
|  | M | 0.56  (0.14) | 0.058  (0.026) | 0.0067  (0.0033) | 6–8 |

Mean on top, (SD) below. Gene expression measured by qPCR in neocortical tissue and expressed as fold change relative to WT + Vehicle. Glial immunoreactivity quantified as percent area in neocortex of 15-month-old mice. Sample sizes for IHC vary by marker due to tissue availability (range shown). *Gfap*, glial fibrillary acidic protein; *Lcn2*, lipocalin-2; *Ptx3*, pentraxin-3. SAA, hAPP^SAA^ KI; WT, hAPP^WT^ KI.
