# Supplementary material for: Glial cytokine modulation improves sleep and circadian disruption in female SAA knock‐in mice of Alzheimer's‐related pathology
Source: Alzheimers Dement. 2026 Mar 30;22(4):e71314. doi: 10.1002/alz.71314 (PMC13140929; doi:10.1002/alz.71314)
Supplement: Supplementary file 3 — Supporting Information [file ALZ-22-e71314-s002.pdf]

ICMJE DISCLOSURE FORM

**Date:**	January 30, 2026
**Your Name:**	Teresa Macheda
**Manuscript Title:**	Glial cytokine modulation rescues sleep and circadian disruption in SAA knock-in Alzheimer mice
**Manuscript Number:**	ADJ-D-25-03525

In the interest of transparency, we ask you to disclose all relationships/activities/interests listed below that are related to the content of your manuscript. "Related" means any relation with for-profit or not-for-profit third parties whose interests may be affected by the content of the manuscript. Disclosure represents a commitment to transparency and does not necessarily indicate a bias.

#	Item	Entity	Comments
1	All support for the present manuscript (e.g., funding, provision of study materials, medical writing, article processing charges, etc.)	None	
2	Grants or contracts from any entity (if not indicated in item #1 above)	None	
3	Royalties or licenses	None	
4	Consulting fees	None	
5	Payment or honoraria for lectures, presentations, speakers bureaus, manuscript writing or educational events	None	
6	Payment for expert testimony	None	
7	Support for attending meetings and/or travel	None	
8	Patents planned, issued or pending	None	
9	Participation on a Data Safety Monitoring Board or Advisory Board	None	
10	Leadership or fiduciary role in other board, society, committee or advocacy group, paid or unpaid	None	
11	Stock or stock options	None	
12	Receipt of equipment, materials, drugs, medical writing, gifts or other services	None	
13	Other financial or non-financial interests	None	

X I certify that I have answered every question and have not altered the wording of any of the questions on this form.

ICMJE DISCLOSURE FORM

**Date:**	January 30, 2026
**Your Name:**	Margaret R. Hawkins
**Manuscript Title:**	Glial cytokine modulation rescues sleep and circadian disruption in SAA knock-in Alzheimer mice
**Manuscript Number:**	ADJ-D-25-03525

In the interest of transparency, we ask you to disclose all relationships/activities/interests listed below that are related to the content of your manuscript. "Related" means any relation with for-profit or not-for-profit third parties whose interests may be affected by the content of the manuscript. Disclosure represents a commitment to transparency and does not necessarily indicate a bias.

#	Item	Entity	Comments
1	All support for the present manuscript (e.g., funding, provision of study materials, medical writing, article processing charges, etc.)	None	
2	Grants or contracts from any entity (if not indicated in item #1 above)	None	
3	Royalties or licenses	None	
4	Consulting fees	None	
5	Payment or honoraria for lectures, presentations, speakers bureaus, manuscript writing or educational events	None	
6	Payment for expert testimony	None	
7	Support for attending meetings and/or travel	None	
8	Patents planned, issued or pending	None	
9	Participation on a Data Safety Monitoring Board or Advisory Board	None	
10	Leadership or fiduciary role in other board, society, committee or advocacy group, paid or unpaid	None	
11	Stock or stock options	None	
12	Receipt of equipment, materials, drugs, medical writing, gifts or other services	None	
13	Other financial or non-financial interests	None	

X I certify that I have answered every question and have not altered the wording of any of the questions on this form.

ICMJE DISCLOSURE FORM

**Date:**	January 30, 2026
**Your Name:**	Carrie E. Johnson
**Manuscript Title:**	Glial cytokine modulation rescues sleep and circadian disruption in SAA knock-in Alzheimer mice
**Manuscript Number:**	ADJ-D-25-03525

In the interest of transparency, we ask you to disclose all relationships/activities/interests listed below that are related to the content of your manuscript. "Related" means any relation with for-profit or not-for-profit third parties whose interests may be affected by the content of the manuscript. Disclosure represents a commitment to transparency and does not necessarily indicate a bias.

#	Item	Entity	Comments
1	All support for the present manuscript (e.g., funding, provision of study materials, medical writing, article processing charges, etc.)	None	
2	Grants or contracts from any entity (if not indicated in item #1 above)	None	
3	Royalties or licenses	None	
4	Consulting fees	None	
5	Payment or honoraria for lectures, presentations, speakers bureaus, manuscript writing or educational events	None	
6	Payment for expert testimony	None	
7	Support for attending meetings and/or travel	None	
8	Patents planned, issued or pending	None	
9	Participation on a Data Safety Monitoring Board or Advisory Board	None	
10	Leadership or fiduciary role in other board, society, committee or advocacy group, paid or unpaid	None	
11	Stock or stock options	None	
12	Receipt of equipment, materials, drugs, medical writing, gifts or other services	None	
13	Other financial or non-financial interests	None	

X I certify that I have answered every question and have not altered the wording of any of the questions on this form.

ICMJE DISCLOSURE FORM

**Date:**	January 30, 2026
**Your Name:**	Madison G. Lapid
**Manuscript Title:**	Glial cytokine modulation rescues sleep and circadian disruption in SAA knock-in Alzheimer mice
**Manuscript Number:**	ADJ-D-25-03525

In the interest of transparency, we ask you to disclose all relationships/activities/interests listed below that are related to the content of your manuscript. "Related" means any relation with for-profit or not-for-profit third parties whose interests may be affected by the content of the manuscript. Disclosure represents a commitment to transparency and does not necessarily indicate a bias.

#	Item	Entity	Comments
1	All support for the present manuscript (e.g., funding, provision of study materials, medical writing, article processing charges, etc.)	None	
2	Grants or contracts from any entity (if not indicated in item #1 above)	None	
3	Royalties or licenses	None	
4	Consulting fees	None	
5	Payment or honoraria for lectures, presentations, speakers bureaus, manuscript writing or educational events	None	
6	Payment for expert testimony	None	
7	Support for attending meetings and/or travel	None	
8	Patents planned, issued or pending	None	
9	Participation on a Data Safety Monitoring Board or Advisory Board	None	
10	Leadership or fiduciary role in other board, society, committee or advocacy group, paid or unpaid	None	
11	Stock or stock options	None	
12	Receipt of equipment, materials, drugs, medical writing, gifts or other services	None	
13	Other financial or non-financial interests	None	

X I certify that I have answered every question and have not altered the wording of any of the questions on this form.

ICMJE DISCLOSURE FORM

**Date:**	January 30, 2026
**Your Name:**	Haleigh R. Whitlock
**Manuscript Title:**	Glial cytokine modulation rescues sleep and circadian disruption in SAA knock-in Alzheimer mice
**Manuscript Number:**	ADJ-D-25-03525

In the interest of transparency, we ask you to disclose all relationships/activities/interests listed below that are related to the content of your manuscript. "Related" means any relation with for-profit or not-for-profit third parties whose interests may be affected by the content of the manuscript. Disclosure represents a commitment to transparency and does not necessarily indicate a bias.

#	Item	Entity	Comments
1	All support for the present manuscript (e.g., funding, provision of study materials, medical writing, article processing charges, etc.)	None	
2	Grants or contracts from any entity (if not indicated in item #1 above)	None	
3	Royalties or licenses	None	
4	Consulting fees	None	
5	Payment or honoraria for lectures, presentations, speakers bureaus, manuscript writing or educational events	None	
6	Payment for expert testimony	None	
7	Support for attending meetings and/or travel	None	
8	Patents planned, issued or pending	None	
9	Participation on a Data Safety Monitoring Board or Advisory Board	None	
10	Leadership or fiduciary role in other board, society, committee or advocacy group, paid or unpaid	None	
11	Stock or stock options	None	
12	Receipt of equipment, materials, drugs, medical writing, gifts or other services	None	
13	Other financial or non-financial interests	None	

X I certify that I have answered every question and have not altered the wording of any of the questions on this form.

ICMJE DISCLOSURE FORM

**Date:**	January 30, 2026
**Your Name:**	Savannah M. Shepard
**Manuscript Title:**	Glial cytokine modulation rescues sleep and circadian disruption in SAA knock-in Alzheimer mice
**Manuscript Number:**	ADJ-D-25-03525

In the interest of transparency, we ask you to disclose all relationships/activities/interests listed below that are related to the content of your manuscript. "Related" means any relation with for-profit or not-for-profit third parties whose interests may be affected by the content of the manuscript. Disclosure represents a commitment to transparency and does not necessarily indicate a bias.

#	Item	Entity	Comments
1	All support for the present manuscript (e.g., funding, provision of study materials, medical writing, article processing charges, etc.)	None	
2	Grants or contracts from any entity (if not indicated in item #1 above)	None	
3	Royalties or licenses	None	
4	Consulting fees	None	
5	Payment or honoraria for lectures, presentations, speakers bureaus, manuscript writing or educational events	None	
6	Payment for expert testimony	None	
7	Support for attending meetings and/or travel	None	
8	Patents planned, issued or pending	None	
9	Participation on a Data Safety Monitoring Board or Advisory Board	None	
10	Leadership or fiduciary role in other board, society, committee or advocacy group, paid or unpaid	None	
11	Stock or stock options	None	
12	Receipt of equipment, materials, drugs, medical writing, gifts or other services	None	
13	Other financial or non-financial interests	None	

X I certify that I have answered every question and have not altered the wording of any of the questions on this form.

ICMJE DISCLOSURE FORM

**Date:**	January 30, 2026
**Your Name:**	MaKayla F. Cox
**Manuscript Title:**	Glial cytokine modulation rescues sleep and circadian disruption in SAA knock-in Alzheimer mice
**Manuscript Number:**	ADJ-D-25-03525

In the interest of transparency, we ask you to disclose all relationships/activities/interests listed below that are related to the content of your manuscript. "Related" means any relation with for-profit or not-for-profit third parties whose interests may be affected by the content of the manuscript. Disclosure represents a commitment to transparency and does not necessarily indicate a bias.

#	Item	Entity	Comments
1	All support for the present manuscript (e.g., funding, provision of study materials, medical writing, article processing charges, etc.)	None	
2	Grants or contracts from any entity (if not indicated in item #1 above)	None	
3	Royalties or licenses	None	
4	Consulting fees	None	
5	Payment or honoraria for lectures, presentations, speakers bureaus, manuscript writing or educational events	None	
6	Payment for expert testimony	None	
7	Support for attending meetings and/or travel	None	
8	Patents planned, issued or pending	None	
9	Participation on a Data Safety Monitoring Board or Advisory Board	None	
10	Leadership or fiduciary role in other board, society, committee or advocacy group, paid or unpaid	None	
11	Stock or stock options	None	
12	Receipt of equipment, materials, drugs, medical writing, gifts or other services	None	
13	Other financial or non-financial interests	None	

X I certify that I have answered every question and have not altered the wording of any of the questions on this form.

ICMJE DISCLOSURE FORM

**Date:**	January 30, 2026
**Your Name:**	Kelly N. Roberts
**Manuscript Title:**	Glial cytokine modulation rescues sleep and circadian disruption in SAA knock-in Alzheimer mice
**Manuscript Number:**	ADJ-D-25-03525

In the interest of transparency, we ask you to disclose all relationships/activities/interests listed below that are related to the content of your manuscript. "Related" means any relation with for-profit or not-for-profit third parties whose interests may be affected by the content of the manuscript. Disclosure represents a commitment to transparency and does not necessarily indicate a bias.

#	Item	Entity	Comments
1	All support for the present manuscript (e.g., funding, provision of study materials, medical writing, article processing charges, etc.)	None	
2	Grants or contracts from any entity (if not indicated in item #1 above)	None	
3	Royalties or licenses	None	
4	Consulting fees	None	
5	Payment or honoraria for lectures, presentations, speakers bureaus, manuscript writing or educational events	None	
6	Payment for expert testimony	None	
7	Support for attending meetings and/or travel	None	
8	Patents planned, issued or pending	None	
9	Participation on a Data Safety Monitoring Board or Advisory Board	None	
10	Leadership or fiduciary role in other board, society, committee or advocacy group, paid or unpaid	None	
11	Stock or stock options	None	
12	Receipt of equipment, materials, drugs, medical writing, gifts or other services	None	
13	Other financial or non-financial interests	None	

X I certify that I have answered every question and have not altered the wording of any of the questions on this form.

ICMJE DISCLOSURE FORM

**Date:**	January 30, 2026
**Your Name:**	Leke Bytyqi
**Manuscript Title:**	Glial cytokine modulation rescues sleep and circadian disruption in SAA knock-in Alzheimer mice
**Manuscript Number:**	ADJ-D-25-03525

In the interest of transparency, we ask you to disclose all relationships/activities/interests listed below that are related to the content of your manuscript. "Related" means any relation with for-profit or not-for-profit third parties whose interests may be affected by the content of the manuscript. Disclosure represents a commitment to transparency and does not necessarily indicate a bias.

#	Item	Entity	Comments
1	All support for the present manuscript (e.g., funding, provision of study materials, medical writing, article processing charges, etc.)	None	
2	Grants or contracts from any entity (if not indicated in item #1 above)	None	
3	Royalties or licenses	None	
4	Consulting fees	None	
5	Payment or honoraria for lectures, presentations, speakers bureaus, manuscript writing or educational events	None	
6	Payment for expert testimony	None	
7	Support for attending meetings and/or travel	None	
8	Patents planned, issued or pending	None	
9	Participation on a Data Safety Monitoring Board or Advisory Board	None	
10	Leadership or fiduciary role in other board, society, committee or advocacy group, paid or unpaid	None	
11	Stock or stock options	None	
12	Receipt of equipment, materials, drugs, medical writing, gifts or other services	None	
13	Other financial or non-financial interests	None	

X I certify that I have answered every question and have not altered the wording of any of the questions on this form.

ICMJE DISCLOSURE FORM

**Date:**	January 30, 2026
**Your Name:**	Heather M. Hash
**Manuscript Title:**	Glial cytokine modulation rescues sleep and circadian disruption in SAA knock-in Alzheimer mice
**Manuscript Number:**	ADJ-D-25-03525

In the interest of transparency, we ask you to disclose all relationships/activities/interests listed below that are related to the content of your manuscript. "Related" means any relation with for-profit or not-for-profit third parties whose interests may be affected by the content of the manuscript. Disclosure represents a commitment to transparency and does not necessarily indicate a bias.

#	Item	Entity	Comments
1	All support for the present manuscript (e.g., funding, provision of study materials, medical writing, article processing charges, etc.)	None	
2	Grants or contracts from any entity (if not indicated in item #1 above)	None	
3	Royalties or licenses	None	
4	Consulting fees	None	
5	Payment or honoraria for lectures, presentations, speakers bureaus, manuscript writing or educational events	None	
6	Payment for expert testimony	None	
7	Support for attending meetings and/or travel	None	
8	Patents planned, issued or pending	None	
9	Participation on a Data Safety Monitoring Board or Advisory Board	None	
10	Leadership or fiduciary role in other board, society, committee or advocacy group, paid or unpaid	None	
11	Stock or stock options	None	
12	Receipt of equipment, materials, drugs, medical writing, gifts or other services	None	
13	Other financial or non-financial interests	None	

X I certify that I have answered every question and have not altered the wording of any of the questions on this form.

ICMJE DISCLOSURE FORM

**Date:**	January 30, 2026
**Your Name:**	Omar A. Abou El-Ezz
**Manuscript Title:**	Glial cytokine modulation rescues sleep and circadian disruption in SAA knock-in Alzheimer mice
**Manuscript Number:**	ADJ-D-25-03525

In the interest of transparency, we ask you to disclose all relationships/activities/interests listed below that are related to the content of your manuscript. "Related" means any relation with for-profit or not-for-profit third parties whose interests may be affected by the content of the manuscript. Disclosure represents a commitment to transparency and does not necessarily indicate a bias.

#	Item	Entity	Comments
1	All support for the present manuscript (e.g., funding, provision of study materials, medical writing, article processing charges, etc.)	None	
2	Grants or contracts from any entity (if not indicated in item #1 above)	None	
3	Royalties or licenses	None	
4	Consulting fees	None	
5	Payment or honoraria for lectures, presentations, speakers bureaus, manuscript writing or educational events	None	
6	Payment for expert testimony	None	
7	Support for attending meetings and/or travel	None	
8	Patents planned, issued or pending	None	
9	Participation on a Data Safety Monitoring Board or Advisory Board	None	
10	Leadership or fiduciary role in other board, society, committee or advocacy group, paid or unpaid	None	
11	Stock or stock options	None	
12	Receipt of equipment, materials, drugs, medical writing, gifts or other services	None	
13	Other financial or non-financial interests	None	

X I certify that I have answered every question and have not altered the wording of any of the questions on this form.

ICMJE DISCLOSURE FORM

**Date:**	January 30, 2026
**Your Name:**	Mohammed Abou El-Ezz
**Manuscript Title:**	Glial cytokine modulation rescues sleep and circadian disruption in SAA knock-in Alzheimer mice
**Manuscript Number:**	ADJ-D-25-03525

In the interest of transparency, we ask you to disclose all relationships/activities/interests listed below that are related to the content of your manuscript. "Related" means any relation with for-profit or not-for-profit third parties whose interests may be affected by the content of the manuscript. Disclosure represents a commitment to transparency and does not necessarily indicate a bias.

#	Item	Entity	Comments
1	All support for the present manuscript (e.g., funding, provision of study materials, medical writing, article processing charges, etc.)	None	
2	Grants or contracts from any entity (if not indicated in item #1 above)	None	
3	Royalties or licenses	None	
4	Consulting fees	None	
5	Payment or honoraria for lectures, presentations, speakers bureaus, manuscript writing or educational events	None	
6	Payment for expert testimony	None	
7	Support for attending meetings and/or travel	None	
8	Patents planned, issued or pending	None	
9	Participation on a Data Safety Monitoring Board or Advisory Board	None	
10	Leadership or fiduciary role in other board, society, committee or advocacy group, paid or unpaid	None	
11	Stock or stock options	None	
12	Receipt of equipment, materials, drugs, medical writing, gifts or other services	None	
13	Other financial or non-financial interests	None	

X I certify that I have answered every question and have not altered the wording of any of the questions on this form.

ICMJE DISCLOSURE FORM

**Date:**	January 30, 2026
**Your Name:**	Katherina Kohler
**Manuscript Title:**	Glial cytokine modulation rescues sleep and circadian disruption in SAA knock-in Alzheimer mice
**Manuscript Number:**	ADJ-D-25-03525

In the interest of transparency, we ask you to disclose all relationships/activities/interests listed below that are related to the content of your manuscript. "Related" means any relation with for-profit or not-for-profit third parties whose interests may be affected by the content of the manuscript. Disclosure represents a commitment to transparency and does not necessarily indicate a bias.

#	Item	Entity	Comments
1	All support for the present manuscript (e.g., funding, provision of study materials, medical writing, article processing charges, etc.)	None	
2	Grants or contracts from any entity (if not indicated in item #1 above)	None	
3	Royalties or licenses	None	
4	Consulting fees	None	
5	Payment or honoraria for lectures, presentations, speakers bureaus, manuscript writing or educational events	None	
6	Payment for expert testimony	None	
7	Support for attending meetings and/or travel	None	
8	Patents planned, issued or pending	None	
9	Participation on a Data Safety Monitoring Board or Advisory Board	None	
10	Leadership or fiduciary role in other board, society, committee or advocacy group, paid or unpaid	None	
11	Stock or stock options	None	
12	Receipt of equipment, materials, drugs, medical writing, gifts or other services	None	
13	Other financial or non-financial interests	None	

X I certify that I have answered every question and have not altered the wording of any of the questions on this form.

ICMJE DISCLOSURE FORM

**Date:**	January 30, 2026
**Your Name:**	Sridhar Sunderam
**Manuscript Title:**	Glial cytokine modulation rescues sleep and circadian disruption in SAA knock-in Alzheimer mice
**Manuscript Number:**	ADJ-D-25-03525

In the interest of transparency, we ask you to disclose all relationships/activities/interests listed below that are related to the content of your manuscript. "Related" means any relation with for-profit or not-for-profit third parties whose interests may be affected by the content of the manuscript. Disclosure represents a commitment to transparency and does not necessarily indicate a bias.

#	Item	Entity	Comments
1	All support for the present manuscript (e.g., funding, provision of study materials, medical writing, article processing charges, etc.)	None	
2	Grants or contracts from any entity (if not indicated in item #1 above)	None	
3	Royalties or licenses	None	
4	Consulting fees	None	
5	Payment or honoraria for lectures, presentations, speakers bureaus, manuscript writing or educational events	None	
6	Payment for expert testimony	None	
7	Support for attending meetings and/or travel	None	
8	Patents planned, issued or pending	None	
9	Participation on a Data Safety Monitoring Board or Advisory Board	None	
10	Leadership or fiduciary role in other board, society, committee or advocacy group, paid or unpaid	None	
11	Stock or stock options	None	
12	Receipt of equipment, materials, drugs, medical writing, gifts or other services	None	
13	Other financial or non-financial interests	None	

X I certify that I have answered every question and have not altered the wording of any of the questions on this form.

ICMJE DISCLOSURE FORM

**Date:**	January 30, 2026
**Your Name:**	Bruce F. O'Hara
**Manuscript Title:**	Glial cytokine modulation rescues sleep and circadian disruption in SAA knock-in Alzheimer mice
**Manuscript Number:**	ADJ-D-25-03525

In the interest of transparency, we ask you to disclose all relationships/activities/interests listed below that are related to the content of your manuscript. "Related" means any relation with for-profit or not-for-profit third parties whose interests may be affected by the content of the manuscript. Disclosure represents a commitment to transparency and does not necessarily indicate a bias.

#	Item	Entity	Comments
1	All support for the present manuscript (e.g., funding, provision of study materials, medical writing, article processing charges, etc.)	None	
2	Grants or contracts from any entity (if not indicated in item #1 above)	None	
3	Royalties or licenses	None	
4	Consulting fees	None	
5	Payment or honoraria for lectures, presentations, speakers bureaus, manuscript writing or educational events	None	
6	Payment for expert testimony	None	
7	Support for attending meetings and/or travel	None	
8	Patents planned, issued or pending	None	
9	Participation on a Data Safety Monitoring Board or Advisory Board	None	
10	Leadership or fiduciary role in other board, society, committee or advocacy group, paid or unpaid	None	
11	Stock or stock options	Signal Solutions LLC	Co-founder and co-owner; manufactures PiezoSleep system used in this study
12	Receipt of equipment, materials, drugs, medical writing, gifts or other services	None	
13	Other financial or non-financial interests	None	

X I certify that I have answered every question and have not altered the wording of any of the questions on this form.

ICMJE DISCLOSURE FORM

**Date:**	January 30, 2026
**Your Name:**	Linda J. Van Eldik
**Manuscript Title:**	Glial cytokine modulation rescues sleep and circadian disruption in SAA knock-in Alzheimer mice
**Manuscript Number:**	ADJ-D-25-03525

In the interest of transparency, we ask you to disclose all relationships/activities/interests listed below that are related to the content of your manuscript. "Related" means any relation with for-profit or not-for-profit third parties whose interests may be affected by the content of the manuscript. Disclosure represents a commitment to transparency and does not necessarily indicate a bias.

#	Item	Entity	Comments
1	All support for the present manuscript (e.g., funding, provision of study materials, medical writing, article processing charges, etc.)	None	
2	Grants or contracts from any entity (if not indicated in item #1 above)	None	
3	Royalties or licenses	None	
4	Consulting fees	None	
5	Payment or honoraria for lectures, presentations, speakers bureaus, manuscript writing or educational events	None	
6	Payment for expert testimony	None	
7	Support for attending meetings and/or travel	None	
8	Patents planned, issued or pending	MW151 patents	Inventor on patents covering MW151
9	Participation on a Data Safety Monitoring Board or Advisory Board	None	
10	Leadership or fiduciary role in other board, society, committee or advocacy group, paid or unpaid	None	
11	Stock or stock options	ImmunoChem Therapeutics, LLC	Scientific founder
12	Receipt of equipment, materials, drugs, medical writing, gifts or other services	None	
13	Other financial or non-financial interests	None	

X I certify that I have answered every question and have not altered the wording of any of the questions on this form.

ICMJE DISCLOSURE FORM

**Date:**	January 30, 2026
**Your Name:**	Michael P. Murphy
**Manuscript Title:**	Glial cytokine modulation rescues sleep and circadian disruption in SAA knock-in Alzheimer mice
**Manuscript Number:**	ADJ-D-25-03525

In the interest of transparency, we ask you to disclose all relationships/activities/interests listed below that are related to the content of your manuscript. "Related" means any relation with for-profit or not-for-profit third parties whose interests may be affected by the content of the manuscript. Disclosure represents a commitment to transparency and does not necessarily indicate a bias.

#	Item	Entity	Comments
1	All support for the present manuscript (e.g., funding, provision of study materials, medical writing, article processing charges, etc.)	None	
2	Grants or contracts from any entity (if not indicated in item #1 above)	None	
3	Royalties or licenses	None	
4	Consulting fees	None	
5	Payment or honoraria for lectures, presentations, speakers bureaus, manuscript writing or educational events	None	
6	Payment for expert testimony	None	
7	Support for attending meetings and/or travel	None	
8	Patents planned, issued or pending	None	
9	Participation on a Data Safety Monitoring Board or Advisory Board	None	
10	Leadership or fiduciary role in other board, society, committee or advocacy group, paid or unpaid	None	
11	Stock or stock options	None	
12	Receipt of equipment, materials, drugs, medical writing, gifts or other services	None	
13	Other financial or non-financial interests	None	

X I certify that I have answered every question and have not altered the wording of any of the questions on this form.

ICMJE DISCLOSURE FORM

**Date:**	January 30, 2026
**Your Name:**	Marilyn J. Duncan
**Manuscript Title:**	Glial cytokine modulation rescues sleep and circadian disruption in SAA knock-in Alzheimer mice
**Manuscript Number:**	ADJ-D-25-03525

In the interest of transparency, we ask you to disclose all relationships/activities/interests listed below that are related to the content of your manuscript. "Related" means any relation with for-profit or not-for-profit third parties whose interests may be affected by the content of the manuscript. Disclosure represents a commitment to transparency and does not necessarily indicate a bias.

#	Item	Entity	Comments
1	All support for the present manuscript (e.g., funding, provision of study materials, medical writing, article processing charges, etc.)	None	
2	Grants or contracts from any entity (if not indicated in item #1 above)	None	
3	Royalties or licenses	None	
4	Consulting fees	None	
5	Payment or honoraria for lectures, presentations, speakers bureaus, manuscript writing or educational events	None	
6	Payment for expert testimony	None	
7	Support for attending meetings and/or travel	None	
8	Patents planned, issued or pending	None	
9	Participation on a Data Safety Monitoring Board or Advisory Board	None	
10	Leadership or fiduciary role in other board, society, committee or advocacy group, paid or unpaid	None	
11	Stock or stock options	None	
12	Receipt of equipment, materials, drugs, medical writing, gifts or other services	None	
13	Other financial or non-financial interests	None	

X I certify that I have answered every question and have not altered the wording of any of the questions on this form.

ICMJE DISCLOSURE FORM

**Date:**	January 30, 2026
**Your Name:**	Adam D. Bachstetter
**Manuscript Title:**	Glial cytokine modulation rescues sleep and circadian disruption in SAA knock-in Alzheimer mice
**Manuscript Number:**	ADJ-D-25-03525

In the interest of transparency, we ask you to disclose all relationships/activities/interests listed below that are related to the content of your manuscript. "Related" means any relation with for-profit or not-for-profit third parties whose interests may be affected by the content of the manuscript. Disclosure represents a commitment to transparency and does not necessarily indicate a bias.

#	Item	Entity	Comments
1	All support for the present manuscript (e.g., funding, provision of study materials, medical writing, article processing charges, etc.)	None	
2	Grants or contracts from any entity (if not indicated in item #1 above)	None	
3	Royalties or licenses	None	
4	Consulting fees	None	
5	Payment or honoraria for lectures, presentations, speakers bureaus, manuscript writing or educational events	None	
6	Payment for expert testimony	None	
7	Support for attending meetings and/or travel	None	
8	Patents planned, issued or pending	None	
9	Participation on a Data Safety Monitoring Board or Advisory Board	None	
10	Leadership or fiduciary role in other board, society, committee or advocacy group, paid or unpaid	None	
11	Stock or stock options	None	
12	Receipt of equipment, materials, drugs, medical writing, gifts or other services	None	
13	Other financial or non-financial interests	None	

X I certify that I have answered every question and have not altered the wording of any of the questions on this form.
